# Why has Japan become the world’s most long-lived country: insights from a food and nutrition perspective

**DOI:** 10.1038/s41430-020-0677-5

**Published:** 2020-07-13

**Authors:** Shoichiro Tsugane

**Affiliations:** Center for Public Health Sciences, National Cancer Center, 5-1-1 Tsukiji, Chuo-ku, Tokyo 104-0045 Japan

**Keywords:** Diseases, Risk factors

## Abstract

In an international comparison of recent mortality statistics among G7 countries, Japan had the longest average life expectancy, primarily due to remarkably low mortality rates from ischemic heart disease and cancer (particularly breast and prostate). As recently as the 1960s, life expectancy in Japan was the shortest among the G7 countries, owing to relatively high mortality from cerebrovascular disease—particularly intracerebral hemorrhage—and stomach cancer. Mortality rates for these diseases subsequently decreased significantly while the already low rates for ischemic heart disease and cancer also decreased, resulting in Japanese life expectancy becoming the longest. The low mortality rates from ischemic heart disease and cancer are thought to reflect the low prevalence of obesity in Japan; low intake of red meat, specifically saturated fatty acids; and high intakes of fish, specifically n-3 polyunsaturated fatty acids, plant foods such as soybeans, and nonsugar-sweetened beverages such as green tea. The decreasing mortality rates from cerebrovascular disease are thought to reflect the increases in animal foods, milk, and dairy products and consequently in saturated fatty acids and calcium, together with a decrease in salt intake which may have led to a decrease in blood pressure. This decrease in salt and highly salted foods also seems to account for the decrease in stomach cancer. The typical Japanese diet as characterized by plant food and fish as well as modest Westernized diet such as meat, milk and dairy products might be associated with longevity in Japan.

## International comparison and annual trends of life expectancy and mortality

### International comparison

Recent mortality-related statistics for the group of seven (G7) countries (Canada, France, Germany, Italy, Japan, United Kingdom (UK), and United States (US) in alphabetical order) from a World Health Organization (WHO) database are shown in Table 1 [1, 2]. Life expectancy and healthy life expectancy are both longest in Japan, in both men and women; longevity is particularly high in women. Age-standard mortality rate is also the lowest, at about two-thirds that of the US. By cause of death, the lowest mortalities from cancer (in particular, breast and prostate cancer) and ischemic heart disease are notable. In contrast, mortalities from cerebrovascular and infectious respiratory disease are relatively high.

**Table 1:** Mortality statistics in selected countries.

	Canada	France	Germany	Italy	Japan	UK	US
Life expectancy at birth (years) in 2016^a^							
Men	80.9	80.1	78.7	80.5	**81.1**	76.0	79.7
Women	84.7	85.7	83.3	84.9	**87.1**	81.0	83.2
Healthy life expectancy (HALE) at birth (years) in 2016^a^							
Men	72.0	71.8	70.2	72.0	**72.6**	66.9	70.9
Women	74.3	74.9	73.0	74.3	**76.9**	70.1	72.9
Age-standardized death rates per 100,000 world standard population in 2016^b^							
All causes	341	338	409	343	**299**	390	493
Cancer	111	125	120	113	**103**	122	114
Stomach	3	4	5	7	**13**	4	3
Colon and rectum	13	13	13	13	**14**	13	11
Lung	30	28	26	23	**20**	27	28
Breast	9	11	10	10	**5**	10	9
Prostate	5	5	6	4	**3**	8	6
Cardiovascular diseases	76	71	132	103	**73**	91	133
Ischemic Heart Diseases	46	31	73	51	**32**	48	79
Stroke	15	17	22	27	**26**	22	23
Respiratory infections	8	8	9	6	**24**	19	11

Bold values are focusing on Japan.

^a^*WHO:* life expectancy and healthy life expectancy data by country (http://apps.who.int/gho/data/node.main.SDG2016LEX?lang=en).

^b^*WHO:* Global Health Estimates 2016: deaths by cause, age, sex, by country and by region, 2000–2016. (https://www.who.int/healthinfo/global_burden_disease/estimates/en/).

Since 1981, the leading cause of death in Japan has been cancer, which accounted for 27% of total deaths in 2018, followed by heart disease at 15% [3]. The recent longevity of Japanese is due to the low mortality rate of these diseases, which account for nearly half of total deaths.

### Annual trends

**Fig. 1:**
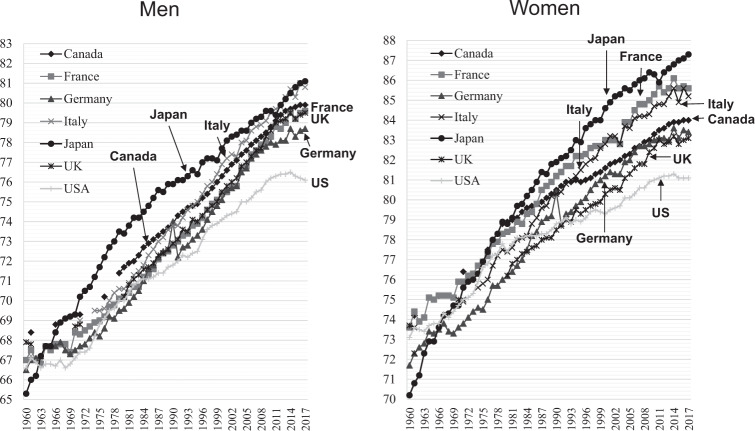
Annual trends of life expectancy at birth (years) in selected countries. The figure was prepared by the author using datafrom “OECD Health Statistics 2019” (https://www.oecd.org/health/health-data.htm).

Figure 1 shows changes in life expectancy in the G7 countries according to health statistics generated by the Organization for Economic Cooperation and Development (OECD) [4]. Whereas Japan had the shortest life expectancy in the early 1960s, Japanese men had the longest in the late 1960s and women in the mid-1970s. These rankings have been maintained despite increases in life expectancy in the other countries. Average life expectancy in Japan in 2016 was 81 in males and 87 in females, a record high. Women have enjoyed world-leading longevity since the 1980s.

**Fig. 2:**
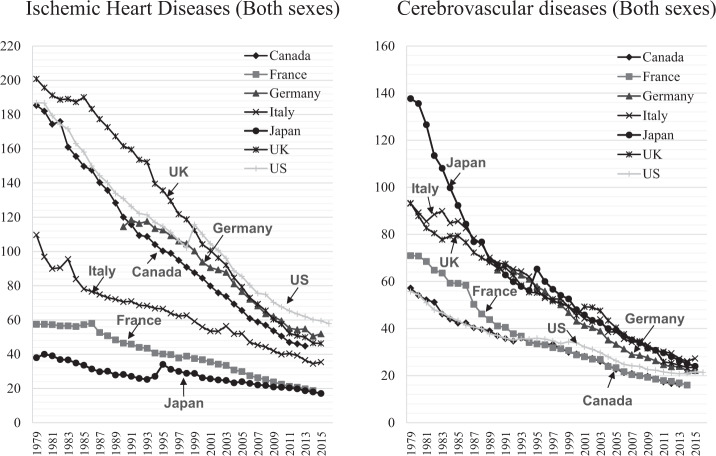
Annual trends in age-standardized circulatory diseases mortality rates per 100,000 world standard population in selected countries. The figure was prepared by the author using datafrom “WHO Mortality Database” (http://apps.who.int/healthinfo/statistics/mortality/whodpms/).

**Fig. 3:**
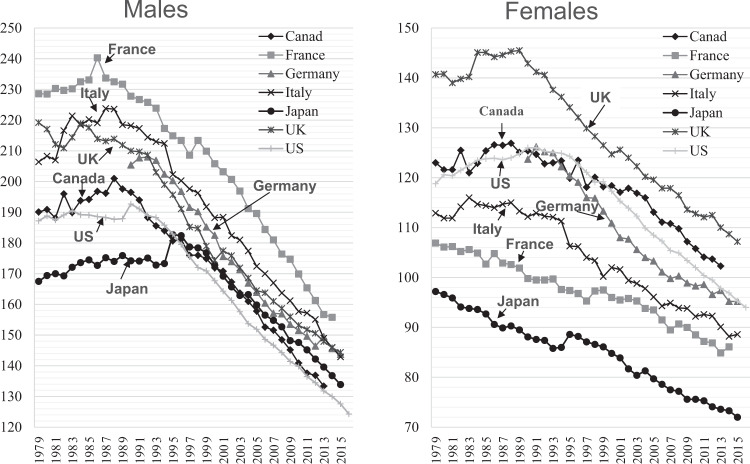
Annual trends in age-standardized cancer mortality rates per 100,000 world standard population in selected countries. The figure was prepared by the author using datafrom “WHO Mortality Database” (http://apps.who.int/healthinfo/statistics/mortality/whodpms/).

Time-course trends in major causes of death according to the WHO Mortality Database [5] show that age-adjusted mortality rates for ischemic heart disease, cerebrovascular disease, and cancer are steadily decreasing in all countries, and that this has resulted in an increase in life expectancy worldwide (Figs. 2, 3). In Japan, mortality from ischemic heart disease and cancer was originally low, while that from cerebrovascular disease—which was extremely high—has steadily declined to a level comparable with that of Western countries. This pattern greatly contributed to Japan’s achieving the world’s highest life expectancy in the 1980s.

Today, ischemic heart disease and cancer in women continue to decline, and remain at the lowest levels. In men, in contrast, cancer mortality was lowest but rose until the mid-1990s, and then began to decline. Lowest levels are now found in US and Canadian men, whose levels have been declining since the 1980s.

**Fig. 4:**
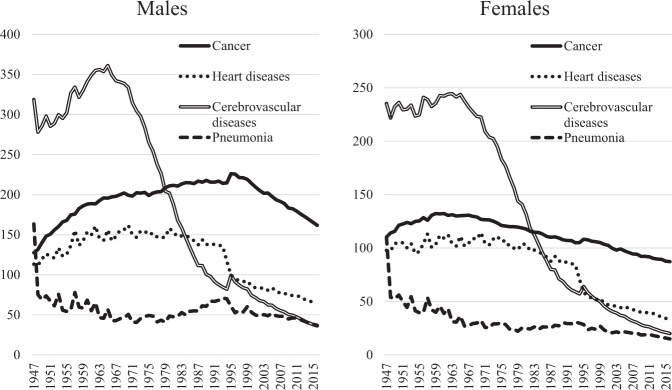
Annual trends in age-standardized mortality rate per 100,000 Japan 1985 model population for leading causes of death. The figure was prepared by the author using datafrom “Vital Statistics, Ministry of Health, Labour and Welfare” (https://www.e-stat.go.jp/).

Although the increase in life expectancy after World War II is in large part due to the dramatic decrease in infant mortality (30.7 per 1000 live births in 1960 vs 2.0 in 2016) in Japan, the steady increase after the war has been attributed to reduced mortality from major causes in adulthood. Looking at annual trends in age-adjusted mortality (1985 model Japanese population) by cause of death (Fig. 4), there was a marked decrease in infectious diseases such as pneumonia and tuberculosis after the war, followed by a decrease in cerebrovascular disease mortality that is characterized by a sharp decline from a peak in the mid-1960s. In addition, heart disease and women’s cancer have been on a gradual decline, while men’s cancer began to decline in the mid-1990s.

The decrease in cerebrovascular disease mortality can be largely explained by the decrease in that due to cerebral hemorrhage, which was dominant around 1950 (Supplementary Fig. 1). Cerebral infarction also increased after the war, but has been decreasing since the mid-1970s. As for cancer mortality (Supplementary Fig. 2), cancers of the stomach and uterus decreased consistently after the war, while liver cancer increased at this time but then declined from the mid-1990s. On the other hand, so-called Western-type cancers such as colon, lung, pancreas, prostate, ovary, and breast tended to increase after the war but have declined since the mid-1990s, except for breast cancer, which until recently continued to increase.

## International comparison and annual trends of major risk factors and dietary factors

### Major risk factors

The prevalence of major risk factors for noncommunicable diseases in G7 countries is shown in Table 2 [6]. Daily cigarette smoking is most prevalent in Japanese men but lowest in Japanese women. Meanwhile, the prevalence of hypertension (SBP ≥ 140 or DBP ≥ 90) is intermediate in both sexes compared with other countries while that of obesity and body mass index is markedly lower.

**Table 2:** Prevalence of major risk factors in selected countries^a^.

Risk factors	Year	Canada	France	Germany	Italy	Japan	UK	US
Men								
Daily cigarette smoking, 15+ years, age standardized (%)	2013	13.7	22.3	25.9	24.5	**28.6**	21.1	16.1
Raised blood pressure (SBP ≧ 140 or DBP ≧ 90), 18+ years, age standardized (%)	2015	15.6	27.7	24.3	25.2	**22.5**	17.9	15.3
Obesity (BMI ≧ 30), 18+ years, age standardized (%)	2016	29.5	22.0	24.2	20.1	**4.8**	26.9	35.5
Mean body mass index trends, age standardized (kg/m²)	2016	27.3	25.9	27.3	26.5	**23.6**	27.3	28.8
Women								
Daily cigarette smoking, 15+ years, age standardized (%)	2013	9.7	18.1	20.4	18.1	**8.4**	19.5	12.6
Raised blood pressure (SBP ≧ 140 or DBP ≧ 90), 18+ years, age standardized (%)	2015	10.8	16.4	15.5	17.1	**12.6**	12.4	10.5
Obesity (BMI ≧ 30), 18+ years, age standardized (%)	2016	29.3	21.1	20.4	19.5	**3.7**	28.6	37.0
Mean body mass index trends, age standardized (kg/m²)	2016	26.6	24.2	25.8	24.7	**21.8**	27.0	28.9

Bold values are focusing on Japan.

^a^*WHO:* Global Health Observatory data repository: noncommunicale diseases, risk factors (http://apps.who.int/gho/data/node.main.A867?lang=en).

Japanese tobacco consumption increased sharply beginning in 1920 and, after a temporary decline during World War II, peaked in the mid-1970s and has since steadily declined [7]. Smoking rates in men of around 80% in 1970 now compare with around 30% in 2013. In contrast, rates in women have remained constantly low, at around 15% and 8%, respectively [4] (Supplementary Fig. 3).

### Dietary factors

Table 3 shows food supply (food supply quantity) in G7 countries from a database of the Food and Agriculture Organization (FAO) of the United Nations [8]. Among characteristics, Japan consumes less meat (particularly red meat such as beef), milk and dairy products, sugar and sweeteners, and fruits and potatoes, but more fish and seafood, rice, soybeans, and tea (mostly green tea).

**Table 3:** Food supply quantity (kg/capita/year) in selected countries in 2013^a^.

Item	Canada	France	Germany	Italy	Japan	UK	US
Livestock and fish							
Meat	90.75	86.76	85.94	84.04	**49.45**	81.48	115.13
Beef	30.25	23.81	13.16	18.60	**9.15**	18.12	36.24
Pork	22.81	33.05	51.81	40.28	**20.62**	25.79	27.64
Poultry	36.68	22.93	17.75	18.61	**19.42**	31.55	50.01
Fish, seafood	22.52	33.48	12.56	25.08	**48.60**	20.76	21.51
Milk—excluding butter	187.77	241.31	258.70	246.88	**72.06**	232.20	254.69
Crops							
Cereals—excluding beer	119.37	127.24	111.11	158.17	**113.44**	115.85	105.64
Rice (milled equivalent)	12.65	4.88	3.34	5.74	**59.85**	6.39	6.88
Starchy roots	73.49	53.95	61.46	38.43	**30.79**	104.05	56.14
Potatoes and products	71.07	53.79	61.46	38.16	**20.95**	103.86	51.88
Sugar & sweeteners	48.26	39.22	48.49	32.12	**27.08**	41.28	63.76
Fruits—excluding wine	135.65	114.34	88.46	139.79	**52.85**	127.41	104.53
Vegetables	108.47	97.32	92.91	128.87	**102.29**	96.99	113.96
Soyabeans	0.94	0.05	0.88	0.01	**7.34**	0.05	0.04
Tea (including mate)	0.45	0.27	0.71	0.14	**0.95**	1.84	0.52

Bold values are focusing on Japan.

^a^FAO: FAOSTAT (Food balance data) (http://www.fao.org/faostat/en/#home).

**Fig. 5:**
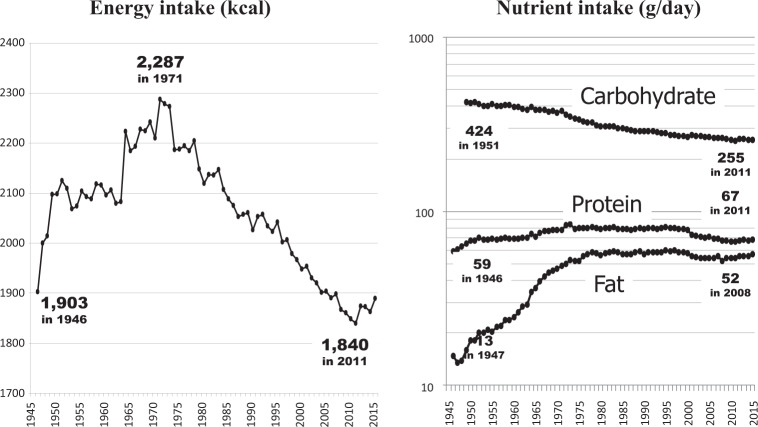
Annual trend of average energy and nutrient intakes in Japan. The figure was prepared by the author using datafrom “National Health and Nutrition Survey, Ministry of Health, Labour and Welfare” (https://www.mhlw.go.jp/bunya/kenkou/kenkou_eiyou_chousa.html).

Looking at changes in energy intake from the National Health and Nutrition Survey [9] (Fig. 5, left), intake was 1903 kcal per person in 1946, the beginning of the postwar period. It then surged to 2125 kcal in 1951, remained flat for a while, then began to increase again during the period of high economic growth, peaking at 2287 kcal in 1971. In response to the end of high economic growth following the 1973 oil crisis, energy intake also continued to decline, reaching a postwar low of 1840 kcal in 2011.

Breaking down the changes in energy intake by the three macronutrients of carbohydrates, fats, and proteins (Fig. 5, right) reveals qualitative changes in the diet of Japanese people. The postwar increase in energy intake was mainly due to an increase in the intake of fasts and proteins from animal foods. In contrast, the intake of carbohydrates continued to decrease throughout this period, mainly due to a decrease in the intake of rice, a staple of the Japanese diet. The increase in fat intake after the war is due to a significant increase in the intake of meat, milk, and dairy products.

Energy intake depends on the balance with the amount of physical activity. The recent decline in energy intake in Japan may represent a response to the decrease in physical activity following widespread worksite automation and automobile use. As a result, men—whose BMI increased after the war but whose working environment significantly improved—continued to gain weight (although balanced in recent years and, most recently, on a declining trend). Among women, a high percentage of whom are full-time housewives, BMI has tended to decrease, some ages excluded, in parallel with the decrease in energy intake (Supplementary Fig. 4). Although BMI has tended to increase in men, it nevertheless remains significantly much lower than in Western countries (Supplementary Fig. 5).

Japan has an international reputation for high salt intake, levels have steadily decreased, from 14.5 g in 1973 to 9.5 g in 2017 [9]. Levels were estimated to be even higher before 1973.

## Why longevity in Japan: a perspective from international comparison

The dietary patterns, as characterized by low intake of red meat, high intakes of fish, plant foods, and nonsugar-sweetened beverages, are thought to be linked to relatively low mortality from cancer and ischemic heart disease and low prevalence of obesity, as follows.

### Red meat and fish

The dietary pattern of lower red meat, milk and dairy products, and higher fish and seafoods results in the lower consumption of saturated fatty acids and higher consumption of n-3 marine polyunsaturated fatty acids in Japan. Dietary intake of saturated fatty acids is associated with increased risk of ischemic heart disease, versus a decreased risk of cerebrovascular disease [10, 11]. In addition, dietary intake of n-3 marine polyunsaturated fatty acids is inversely associated with the risk of ischemic heart disease [12, 13]. Japan’s lower red meat and higher fish consumption may be related to its relatively low mortality from ischemic heart disease, but high mortality from cerebrovascular disease.

### Soybeans and nonstarchy vegetables

Soybeans are mostly consumed in Asian countries, including Japan, and are the sole source of isoflavones, which are known to have anticancer [14] and anticardiovascular [15] effects. Isoflavone intake in the amounts consumed in Asian populations is associated with lower risks of breast [16, 17] and prostate cancer [18, 19]. The relatively higher soy intake may account for the low breast and prostate cancer mortality in Japan. Soy and isoflavone intake have also been inversely associated with risk of cardiovascular diseases, especially cerebral and myocardial infarctions [20, 21]. In a prospective study, we showed that intake of fermented soy products was inversely associated with total and cardiovascular mortalities [22]. Soybeans are also major source of plant protein. In another prospective study, we also showed that higher plant protein intake was associated with lower total and cardiovascular disease mortalities, and that isocaloric substitution of 3% of energy from plant protein for red meat protein was associated with lower total, cancer and cardiovascular disease mortalities [23]. This higher intake of plant protein may also be related to Japanese longevity.

### Less sugar and nonsweetened green tea

Low consumption of sugar sweeteners, and potatoes, and high consumption of green tea (which is not generally sweetened with sugar) may be partly related to a globally lower prevalence of obesity and lower rates of obesity-related diseases such as ischemic heart disease and breast cancer [24, 25]. Our prospective study showed that intake of green tea was inversely associated with all-cause mortality and cardiovascular mortality [26, 27].

### Dietary diversity

In addition, Japanese tend to consume a variety of foods such as grain, vegetable, fruit, fish and meat, and milk dishes, and this dietary pattern might be partly related to Japanese longevity. Our prospective study showed that dietary diversity [28] and adherence to the Japanese Food Guide Spinning Top that encourages the balanced diet [29] were inversely associated with all-cause mortality.

## Why the change to longevity: a perspective from annual trends in Japan

Considering the changes in average life expectancy and mortality of Japanese together with their changes in food and nutrition intake, it is clear that the improvement in nutritional status after the war significantly reduced mortality from infectious diseases such as tuberculosis and pneumonia, and cerebral hemorrhage. This improvement can be considered to have produced a continuous extension of average life expectancy. The suppressed severity of infectious disease and faster recovery in well-nourished persons is well known. With cerebrovascular disease, the increased risk of intracerebral hemorrhage, in which blood vessels rupture, is increased in the face of insufficient cholesterol, an important constituent of the blood vessel wall. The increase in animal foods, milk and dairy products, and consequently in saturated fatty acids, strengthened blood vessel walls; and calcium, together with the decrease in salt intake and spread of antihypertensive drugs, led to decreases in blood pressure and consequent cerebrovascular disease [30–32]. Further, the decreased intake of salt and highly salted foods appeared to have produced a decrease in stomach cancer [33, 34].

On the other hand, if the increase in animal fats and proteins improved nutritional status, it is also presumed to have produced increases in cerebral infarction and ischemic heart diseases, diabetes, and so-called Western-type cancers, such as the colorectum, pancreas, prostate, ovary, and breast. Risk of these diseases is in fact known to increase with overnutrition and the resulting obesity, lack of exercise, and consumption of red meat such as beef, pork, and mutton [35, 36]. However, fat and protein intakes leveled off in the mid-1970s, followed by a decrease in energy intake (Fig. 5). As if linked to this, first, the increase in cerebral infarction stopped and incidence began to decrease and, after a time lag of several decades, the so-called Western-type cancers tended to flat-line or decrease. Cancer takes a long time to develop, and there is a substantial time lag between changes in causal factors and changes in incidence.

From the above, the westernization of diet—an increase in nutrients such as energy, an increase in animal foods, and decrease in salt intake—can be considered to have generally made postwar Japanese people healthier. The convergence of westernization—first detected after World War II, began accelerating in the 1970s due to the economic situation. At this time, the consumption of seafood, plant proteins such as soybeans, and cereals and vegetables were higher than in Western countries, and a lower fat energy ratio (National Health and Nutrition Survey 2017, adult average: 27.4%) has been maintained. Although BMI has tended to increase in men, it nevertheless remains significantly much lower than in Western countries, where obesity is a major health problem (Supplementary Fig. 5).

The decreases in cancers of the stomach, uterus and liver are largely due to decreases in persistent infection with *Helicobacter pylori*, human papilloma virus, and hepatitis viruses, respectively, which are essential risk factors for these cancers [37]. Because smoking accounts for about 30% of male cancer mortality [37], it is speculated that the decline in male age-adjusted cancer mortality, which began in the mid-1990s represents a time lag of about 20 years from the 1970 peak in tobacco consumption. On the other hand, in Europe and North America, the decline in tobacco consumption and smoking rates began about 10 years earlier, so it is estimated that the decrease in age-adjusted mortality rate of cancer in these countries began about 10 years earlier than in Japan.

## Conclusion

Japanese people have remarkably low mortality rates from ischemic heart disease and cancer (particularly breast and prostate), and relatively high rates from cerebrovascular disease and respiratory infection. The world’s longest life expectancy is due to a significant decrease in mortality from infectious diseases, cerebrovascular disease, and pneumonia, which were high in the past, while keeping cancer and ischemic heart disease mortality low.

Low obesity, low intake of saturated fatty acids, and high intakes of marine n-3 polyunsaturated fatty acids, plant foods such as soybeans and nonsugar-sweetened beverages such as green tea may contribute to low cancer and ischemic heart disease mortality. In the past, cerebrovascular disease and stomach cancer mortality rates were extremely high, probably due to a relatively high salt intake and low intake of saturated fatty acids and calcium.

The typical Japanese diet as characterized by plant food and fish as well as modest Westernized diet such as meat, milk, and dairy products might be associated with longevity in Japan.

## Supplementary information

Supplemental Figure 1

Supplemental Figure 2

Supplemental Figure 3

Supplemental Figure 4

Supplemental Figure 5

## References

[1] World Health Organization (WHO). Life expectancy and Healthy life expectancy data by country. http://apps.who.int/gho/data/node.main.SDG2016LEX?lang=en. Accessed March 1, 2020.

[2] World Health Organization (WHO). Global Health Estimates 2016: deaths by cause, age, sex, by country and by region, 2000–2016. https://www.who.int/healthinfo/global_burden_disease/estimates/en/. Accessed March 1, 2020.

[3] Ministry of Health, Labor and Welfare, Japan. Vital statistics. https://www.e-stat.go.jp/. Accessed March 1, 2020.

[4] Organization for Economic Cooperation and Development (OECD). OECD health statistics 2019. https://www.oecd.org/health/health-data.htm. Accessed March 1, 2020.

[5] World Health Organization (WHO). WHO mortality database. http://apps.who.int/healthinfo/statistics/mortality/whodpms/. Accessed March 1, 2020.

[6] World Health Organization (WHO). Global Health Observatory data repository, noncommunicable diseases, risk factors. http://apps.who.int/gho/data/node.main.A867?lang=en. Accessed March 1, 2020.

[7] Japan Health Promotion & Fitness Foundation. Tobacco or health. http://www.health-net.or.jp/tobacco/menu02.html. Accessed March 1, 2020.

[8] Food and Agriculture Organization of the United Nations: FAOSTAT. http://www.fao.org/faostat/en/#home. Accessed March 1, 2020.

[9] Ministry of Health, Labor and Welfare, Japan. National health and nutrition survey. https://www.mhlw.go.jp/bunya/kenkou/kenkou_eiyou_chousa.html. Accessed March 1, 2020.

[10] Yamagishi K, Iso H, Kokubo Y, Saito I, Yatsuya H, Ishihara J (2013). Dietary intake of saturated fatty acids and incident stroke and coronary heart disease in Japanese communities: the JPHC Study. Eur Heart J.

[11] Yamagishi K, Iso H, Tsugane S (2015). Saturated fat intake and cardiovascular disease in Japanese population. J Atheroscler Thromb.

[12] Iso H, Kobayashi M, Ishihara J, Sasaki S, Okada K, Kita Y (2006). Intake of fish and n3 fatty acids and risk of coronary heart disease among Japanese: the Japan Public Health Center-Based (JPHC) Study Cohort I. Circulation.

[13] Sekikawa A, Doyle MF, Kuller LH (2015). Recent findings of long-chain n-3 polyunsaturated fatty acids (LCn-3 PUFAs) on atherosclerosis and coronary heart disease (CHD) contrasting studies in Western countries to Japan. Trends Cardiovasc Med.

[14] Messina MJ, Persky V, Setchell KD, Barnes S (1994). Soy intake and cancer risk: a review of the in vitro and in vivo data. Nutr Cancer.

[15] Sacks FM, Lichtenstein A, Van Horn L, Harris W, Kris-Etherton P, Winston M, American Heart Association Nutrition Committee. (2006). Soy protein, isoflavones, and cardiovascular health: an American Heart Association Science Advisory for professionals from the Nutrition Committee. Circulation.

[16] Yamamoto S, Sobue T, Kobayashi M, Sasaki S, Tsugane S (2003). Soy, isoflavones, and breast cancer risk in Japan. J Natl Cancer Inst.

[17] Xie Q, Chen ML, Qin Y, Zhang QY, Xu HX, Zhou Y (2013). Isoflavone consumption and risk of breast cancer: a dose-response meta-analysis of observational studies. Asia Pac J Clin Nutr.

[18] Kurahashi N, Iwasaki M, Sasazuki S, Otani T, Inoue M, Tsugane S (2007). Soy product and isoflavone consumption in relation to prostate cancer in Japanese men. Cancer Epidemiol Biomark Prev.

[19] Yan L, Spitznagel EL (2009). Soy consumption and prostate cancer risk in men: a revisit of a meta-analysis. Am J Clin Nutr.

[20] Kokubo Y, Iso H, Ishihara J, Okada K, Inoue M, Tsugane S (2007). Association of dietary intake of soy, beans, and isoflavones with risk of cerebral and myocardial infarctions in Japanese populations: the Japan Public Health Center-based (JPHC) study cohort I. Circulation.

[21] Yan Z, Zhang X, Li C, Jiao S, Dong W (2017). Association between consumption of soy and risk of cardiovascular disease: a meta-analysis of observational studies. Eur J Prev Cardiol.

[22] Katagiri R, Sawada N, Goto A, Yamaji T, Iwasaki M, Noda M (2020). Association of soy and fermented soy product intake with total and cause specific mortality: prospective cohort study. BMJ.

[23] Budhathoki S, Sawada N, Iwasaki M, Yamaji T, Goto A, Kotemori A (2019). Association of animal and plant protein intake with all-cause and cause-specific mortality in a Japanese cohort. JAMA Intern Med.

[24] Hruby A, Manson JE, Qi L, Malik VS, Rimm EB, Sun Q (2016). Determinants and consequences of obesity. Am J Public Health.

[25] Malik VS, Popkin BM, Bray GA, Després JP, Hu FB (2010). Sugar-sweetened beverages, obesity, type 2 diabetes mellitus, and cardiovascular disease risk. Circulation.

[26] Saito E, Inoue M, Sawada N, Shimazu T, Yamaji T, Iwasaki M (2015). Association of green tea consumption with mortality due to all causes and major causes of death in a Japanese population: the Japan Public Health Center-based Prospective Study (JPHC Study). Ann Epidemiol.

[27] Abe SK, Saito E, Sawada N, Tsugane S, Ito H, Lin Y (2019). Green tea consumption and mortality in Japanese men and women: a pooled analysis of eight population-based cohort studies in Japan. Eur J Epidemiol.

[28] Kobayashi M, Sasazuki S, Shimazu T, Sawada N, Yamaji T, Iwasaki M (2020). Association of dietary diversity with total mortality and major causes of mortality in the Japanese population: JPHC study. Eur J Clin Nutr.

[29] Kurotani K, Akter S, Kashino I, Goto A, Mizoue T, Noda M (2016). Quality of diet and mortality among Japanese men and women: Japan Public Health Center based prospective study. BMJ.

[30] Shimamoto T, Iso H, Iida M, Komachi Y (1996). Epidemiology of cerebrovascular disease: stroke epidemic in Japan. J Epidemiol.

[31] Yu E, Hu FB (2018). Dairy products, dairy fatty acids, and the prevention of cardiometabolic disease: a review of recent evidence. Curr Atheroscler Rep.

[32] Stamler J, Rose G, Stamler R, Elliott P, Dyer A, Marmot M (1989). INTERSALT study findings. Public health and medical care implications. Hypertension.

[33] Tsugane S (2005). Salt, salted food intake, and risk of gastric cancer: epidemiologic evidence. Cancer Sci.

[34] Joossens JV, Hill MJ, Elliott P, Stamler R, Lesaffre E, Dyer A (1996). Dietary salt, nitrate and stomach cancer mortality in 24 countries. European Cancer Prevention (ECP) and the INTERSALT Cooperative Research Group. Int J Epidemiol.

[35] Willett WC (2008). Overview and perspective in human nutrition. Asia Pac J Clin Nutr.

[36] Key TJ, Bradbury KE, Perez-Cornago A, Sinha R, Tsilidis KK, Tsugane S (2020). Diet, nutrition and cancer risk: what do we know and what is the way forward?. BMJ.

[37] Inoue M, Sawada N, Matsuda T, Iwasaki M, Sasazuki S, Shimazu T (2012). Attributable causes of cancer in Japan in 2005-systematic assessment to estimate current burden of cancer attributable to known preventable risk factors in Japan. Ann Oncol.

