# Supplementary material for: Why has Japan become the world’s most long-lived country: insights from a food and nutrition perspective
Source: Eur J Clin Nutr. 2020 Jul 13;75(6):921–8. doi: 10.1038/s41430-020-0677-5 (PMC8189904; doi:10.1038/s41430-020-0677-5)
Supplement: Supplementary file 1 — Supplemental Figure 1 [file 41430_2020_677_MOESM1_ESM.pptx]

## Slide 1
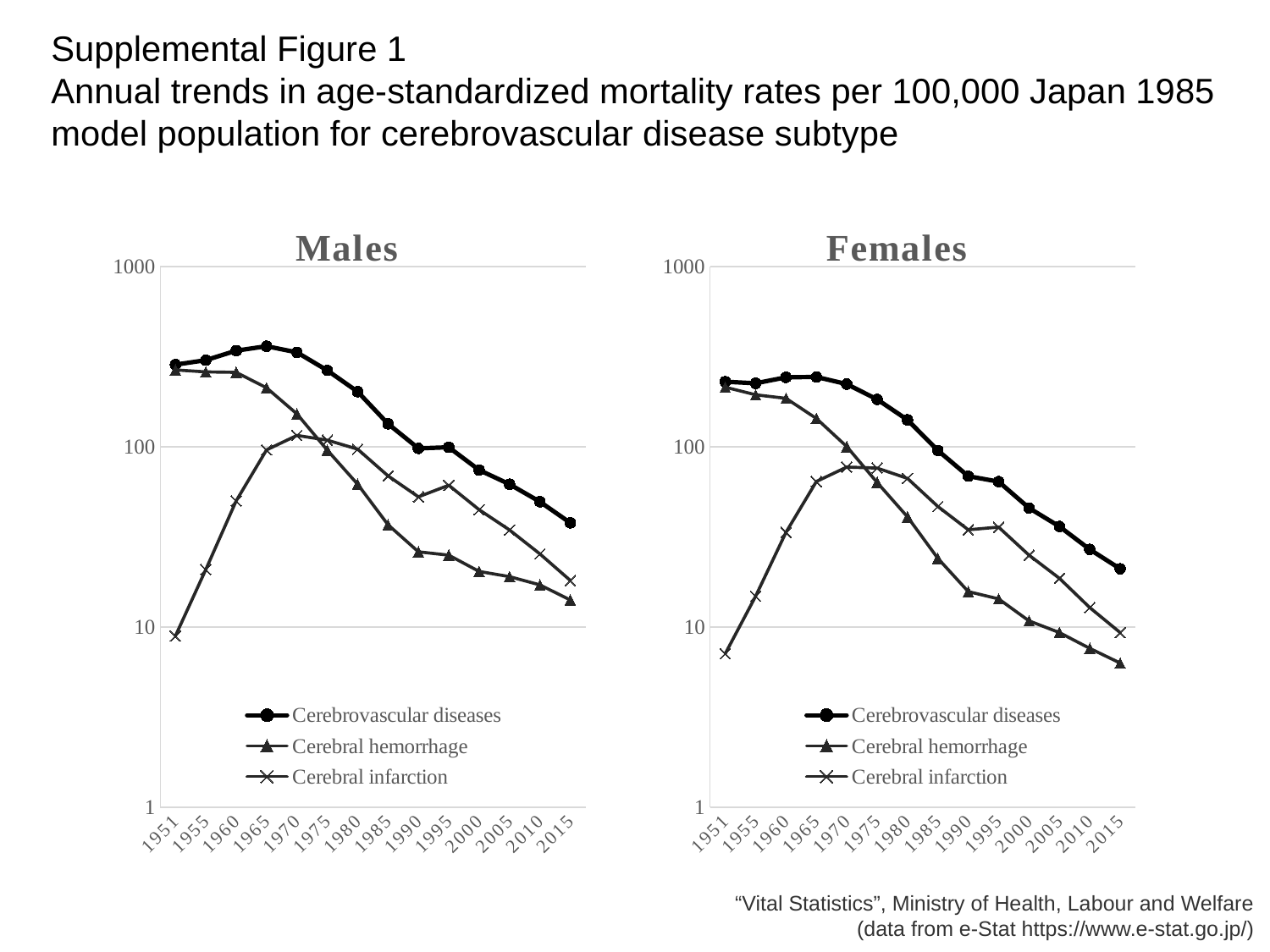

# Supplemental Figure 1 Annual trends in age-standardized mortality rates per 100,000 Japan 1985 model population for cerebrovascular disease subtype
### Chart: Males
| Category | Cerebrovascular diseases | Cerebral hemorrhage | Cerebral infarction |
|---|---|---|---|
| 1951 | 285.3 | 266.7 | 8.9 |
| 1955 | 302.1 | 260.1 | 20.8 |
| 1960 | 341.1 | 258.5 | 50.0 |
| 1965 | 361.0 | 212.2 | 96.0 |
| 1970 | 333.8 | 152.0 | 115.5 |
| 1975 | 265.0 | 95.7 | 108.7 |
| 1980 | 202.0 | 61.9 | 96.9 |
| 1985 | 134.0 | 36.9 | 68.9 |
| 1990 | 97.9 | 26.1 | 52.7 |
| 1995 | 99.3 | 25.0 | 61.1 |
| 2000 | 74.2 | 20.3 | 44.7 |
| 2005 | 61.9 | 19.0 | 34.5 |
| 2010 | 49.5 | 17.1 | 25.4 |
| 2015 | 37.8 | 14.1 | 18.1 |
### Chart: Females
| Category | Cerebrovascular diseases | Cerebral hemorrhage | Cerebral infarction |
|---|---|---|---|
| 1951 | 229.4 | 213.9 | 7.1 |
| 1955 | 224.8 | 194.1 | 14.8 |
| 1960 | 242.7 | 185.5 | 33.5 |
| 1965 | 243.8 | 143.4 | 64.1 |
| 1970 | 222.6 | 100.1 | 77.1 |
| 1975 | 183.0 | 63.3 | 76.1 |
| 1980 | 140.9 | 40.8 | 66.7 |
| 1985 | 95.3 | 24.0 | 46.6 |
| 1990 | 68.6 | 15.7 | 34.6 |
| 1995 | 64.0 | 14.3 | 35.8 |
| 2000 | 45.7 | 10.8 | 25.0 |
| 2005 | 36.1 | 9.3 | 18.6 |
| 2010 | 26.9 | 7.6 | 12.8 |
| 2015 | 21.0 | 6.3 | 9.3 |“Vital Statistics”, Ministry of Health, Labour and Welfare
(data from e-Stat https://www.e-stat.go.jp/)
