# Supplementary material for: Why has Japan become the world’s most long-lived country: insights from a food and nutrition perspective
Source: Eur J Clin Nutr. 2020 Jul 13;75(6):921–8. doi: 10.1038/s41430-020-0677-5 (PMC8189904; doi:10.1038/s41430-020-0677-5)
Supplement: Supplementary file 2 — Supplemental Figure 2 [file 41430_2020_677_MOESM2_ESM.pptx]

## Slide 1
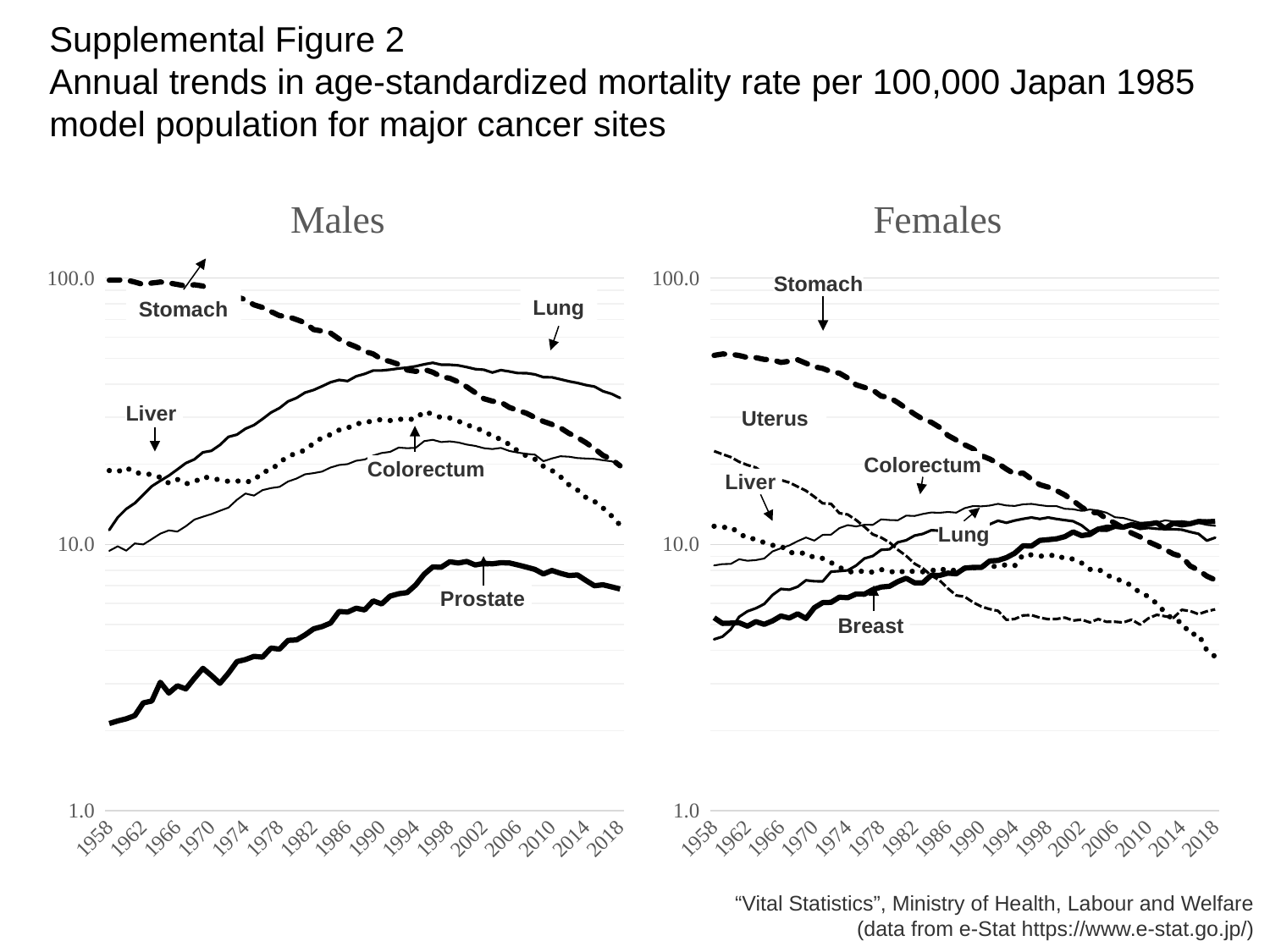

# Supplemental Figure 2 Annual trends in age-standardized mortality rate per 100,000 Japan 1985 model population for major cancer sites
### Chart: Males
| Category | 胃 | 大腸 | 肝臓 | 肺 | 前立腺 |
|---|---|---|---|---|---|
| 1958 | 98.28412892 | 9.4540866906 | 18.948268085 | 11.327859232 | 2.1241519481 |
| 1959 | 98.446460277 | 9.8365190336 | 18.738557375 | 12.634719285 | 2.1728350674 |
| 1960 | 98.455402708 | 9.4727798438 | 19.447873847 | 13.593572089 | 2.2129614511 |
| 1961 | 96.711493769 | 10.078492603 | 18.581683691 | 14.288414462 | 2.274323085 |
| 1962 | 94.671133609 | 9.995212941 | 18.485286039 | 15.377132013 | 2.5396675913 |
| 1963 | 95.88210402 | 10.466637817 | 18.287160916 | 16.539027778 | 2.5820915485000002 |
| 1964 | 96.757334404 | 10.970067449 | 17.866004047 | 17.336271912 | 3.0342686081 |
| 1965 | 95.974496837 | 11.291143657 | 16.982720201 | 18.134208966 | 2.7656752998 |
| 1966 | 94.732672008 | 11.170921451 | 17.552562533 | 19.126672119 | 2.9440732908 |
| 1967 | 93.456324243 | 11.69955983 | 16.893158879 | 20.208066618 | 2.8666459568 |
| 1968 | 94.492751601 | 12.395334676 | 17.149092322 | 20.8731777 | 3.1406492571 |
| 1969 | 93.419691027 | 12.710238474 | 17.961778869 | 22.173830862 | 3.4233601848 |
| 1970 | 88.868613055 | 13.000022314 | 17.706328973 | 22.471513979 | 3.2183923687 |
| 1971 | 87.406242083 | 13.377226788 | 17.476543823 | 23.632380073 | 3.0070945813 |
| 1972 | 87.063785994 | 13.750438796 | 17.25626774 | 25.349252359 | 3.2831588788 |
| 1973 | 85.033779976 | 14.713332814 | 17.325652248 | 25.846209123 | 3.6283057053 |
| 1974 | 83.008797783 | 15.531037283 | 17.127352 | 27.202793777 | 3.6943139056 |
| 1975 | 79.40929604 | 15.257098085 | 17.403187773 | 28.069508511 | 3.7978637776 |
| 1976 | 77.60114724 | 15.989504584 | 18.708052842 | 29.578314681 | 3.7704222768 |
| 1977 | 74.905462285 | 16.278591116 | 19.020147122 | 31.293029864 | 4.0806459888 |
| 1978 | 72.297258713 | 16.454836202 | 20.304185673 | 32.533908561 | 4.0412538423 |
| 1979 | 71.751485553 | 17.232674397 | 21.447713777 | 34.452275451 | 4.3610217109 |
| 1980 | 69.89134606 | 17.679540574 | 21.997624693 | 35.493297229 | 4.3768379585 |
| 1981 | 68.029238999 | 18.340687793 | 22.603635921 | 37.182881477 | 4.5709384771 |
| 1982 | 64.115962516 | 18.524099324 | 23.985986323 | 38.022828521 | 4.8162845794 |
| 1983 | 63.327879734 | 18.783304421 | 25.174889753 | 39.272979817 | 4.9137666177 |
| 1984 | 62.197382367 | 19.450845972 | 25.856057212 | 40.664911374 | 5.065496959 |
| 1985 | 59.097094958 | 19.875373107 | 26.88784722 | 41.478289778 | 5.5976320052 |
| 1986 | 56.895429587 | 20.028895603 | 27.374354578 | 41.081046751 | 5.569292765 |
| 1987 | 55.246809317 | 20.620211275 | 28.397911329 | 42.803202313 | 5.756307442 |
| 1988 | 53.086393727 | 20.841145641 | 28.596873471 | 43.699202707 | 5.6730483801 |
| 1989 | 51.938548643 | 21.555279367 | 29.188936911 | 44.986672873 | 6.1341310178 |
| 1990 | 49.5467401 | 22.051705305 | 29.453144271 | 45.006191661 | 5.9743455354 |
| 1991 | 48.665143318 | 22.266421113 | 29.198571258 | 45.34183435 | 6.3942871737 |
| 1992 | 47.446070413 | 23.116699081 | 29.554462051 | 45.80081703 | 6.5259621489 |
| 1993 | 45.190747864 | 22.97860092 | 29.404956559 | 46.148807477 | 6.5949682546 |
| 1994 | 44.67749094 | 23.060523048 | 29.673603328 | 46.693386214 | 7.0404250008 |
| 1995 | 45.421633589 | 24.439283958405372 | 31.583700934 | 47.472012822 | 7.7308164667 |
| 1996 | 44.340897289 | 24.7148374421156 | 30.788121529 | 48.103937224 | 8.2314604441 |
| 1997 | 42.629831755 | 24.241468251250627 | 29.942710844 | 47.312445751 | 8.2074553266 |
| 1998 | 42.09585082 | 24.379220050374418 | 29.865546309 | 47.294568671 | 8.6061405187 |
| 1999 | 40.763031055 | 24.150950300630992 | 29.043645199 | 47.026241541 | 8.5112757047 |
| 2000 | 39.054335359 | 23.71939109534552 | 28.164924791 | 46.338619373 | 8.6271974886 |
| 2001 | 37.145430124 | 23.44120738705776 | 27.34414688 | 45.557108312 | 8.3662671178 |
| 2002 | 35.263648525 | 22.99193624912277 | 26.725952377 | 45.299958847 | 8.4903040839 |
| 2003 | 34.533739202 | 22.81820795257802 | 25.456124072 | 44.212464338 | 8.4541641545 |
| 2004 | 34.219890373 | 23.041559981793615 | 24.834076273 | 45.150046519 | 8.5324140552 |
| 2005 | 32.697755905 | 22.443074528352057 | 23.716784935 | 44.615783647 | 8.51793613 |
| 2006 | 31.888452167 | 22.12296260340628 | 22.423617058 | 44.003354106 | 8.3722725364 |
| 2007 | 31.105349393 | 21.888874731546785 | 21.509715111 | 43.979689986 | 8.2165460651 |
| 2008 | 29.965760102 | 21.737086664598486 | 20.903874006 | 43.512261734 | 8.0535104973 |
| 2009 | 28.978419991 | 20.540960867639892 | 19.651763086 | 42.468820034 | 7.7444358898 |
| 2010 | 28.248339761 | 21.0327579376526 | 18.965286523 | 42.436780396 | 7.9825398625 |
| 2011 | 27.396932455086457 | 21.435521654060135 | 17.965221685660058 | 41.712913295677694 | 7.786655698009159 |
| 2012 | 26.127886079100517 | 21.352055585731197 | 16.745886598820547 | 40.974448978436506 | 7.636515927121342 |
| 2013 | 25.2 | 21.103923030345637 | 16.0 | 40.416419457671395 | 7.677842891687582 |
| 2014 | 24.1 | 21.02839133956695 | 15.0 | 39.66092051712232 | 7.31781257092784 |
| 2015 | 22.9 | 20.95447801234815 | 14.5 | 39.16473285311138 | 6.992363200070973 |
| 2016 | 21.6 | 20.712369939498863 | 13.7 | 37.61138686984237 | 7.05208686097357 |
| 2017 | 20.9 | 20.5201201507787 | 12.8 | 36.78372037296664 | 6.926152627214146 |
| 2018 | 19.74252374036772 | 19.965222720225064 | 11.815636111347377 | 35.5 | 6.8 |
### Chart: Females
| Category | 胃 | 大腸 | 肝臓 | 肺 | 乳房 | 子宮 |
|---|---|---|---|---|---|---|
| 1958 | 51.300916611 | 8.3337835931 | 11.699521512 | 4.4005786476 | 5.3046473011 | 22.414002697 |
| 1959 | 51.893545823 | 8.4321258833 | 11.581122778 | 4.5022522911 | 5.0508705696 | 21.816350304 |
| 1960 | 51.836995021 | 8.4477062987 | 11.613924981 | 4.7925934727 | 5.061889073 | 21.282835488 |
| 1961 | 51.227311324 | 8.7931600673 | 11.016871417 | 5.3454990854 | 5.0775626633 | 20.434267695 |
| 1962 | 50.366027309 | 8.6796755707 | 10.528444643 | 5.607407772 | 4.9296449081 | 19.865534742 |
| 1963 | 50.298095269 | 8.7297068426 | 10.48274904 | 5.7545504803 | 5.1282056285 | 19.445936389 |
| 1964 | 49.537353769 | 8.8513706909 | 10.147237584 | 5.9690250051 | 5.0103711422 | 18.434151946 |
| 1965 | 49.372412642 | 9.412026581 | 9.9233578679 | 6.4580051342 | 5.160186231 | 18.004141782 |
| 1966 | 48.270146083 | 9.6725976982 | 9.869437087 | 6.8037882638 | 5.3839961802 | 17.457326779 |
| 1967 | 48.749330638 | 9.9070875277 | 9.3250404028 | 6.7551421118 | 5.290514468 | 17.076510621 |
| 1968 | 49.390715653 | 10.284750946 | 9.2788498094 | 6.9326291252 | 5.4871870421 | 16.484715373 |
| 1969 | 47.911979448 | 10.610284403 | 9.2687024267 | 7.3300880104 | 5.2699870148 | 15.912604427 |
| 1970 | 46.457917222 | 10.332133031 | 8.9053899723 | 7.2721727269 | 5.7822649918 | 15.108506024 |
| 1971 | 45.821937848 | 10.859642043 | 8.8640868932 | 7.2621975528 | 6.0431717027 | 14.269582771 |
| 1972 | 44.530772046 | 10.880628856 | 8.5433198437 | 7.8979543195 | 6.0594285476 | 14.204073063 |
| 1973 | 43.984140983 | 11.509899919 | 8.2028153272 | 7.9381481059 | 6.3354839599 | 13.103407395 |
| 1974 | 42.170259431 | 11.81130456 | 7.9246826828 | 7.9881164211 | 6.3051801516 | 12.954004767 |
| 1975 | 39.772132529 | 11.681619618 | 7.8317525275 | 8.3272224576 | 6.5112270797 | 12.35106739 |
| 1976 | 38.873783903 | 11.848983681 | 7.9822121679 | 8.8540928801 | 6.50035541 | 11.641333738 |
| 1977 | 38.008258438 | 11.83917284 | 7.8534680133 | 9.0455708151 | 6.7625517329 | 10.903125706 |
| 1978 | 36.058056738 | 12.419883625 | 8.0424919387 | 9.536630305 | 6.9147278951 | 10.609093472 |
| 1979 | 35.665358408 | 12.348847975 | 7.945652066 | 9.5875433765 | 6.9587452882 | 10.172411477 |
| 1980 | 34.078924822 | 12.322902251 | 7.782031403 | 10.182435701 | 7.2488638787 | 9.5459974062 |
| 1981 | 32.428679144 | 12.827300345 | 8.0187411502 | 10.362207821 | 7.4605608065 | 9.0553807029 |
| 1982 | 30.866419214 | 12.79318121 | 7.8835606599 | 10.788991546 | 7.1752427944 | 8.4661664026 |
| 1983 | 29.534575092 | 13.018269419 | 7.8910357102 | 10.951697944 | 7.174334477 | 8.1280945253 |
| 1984 | 28.777296826 | 13.183390642 | 7.9504663507 | 11.298504715 | 7.6619746821 | 7.6422417196 |
| 1985 | 27.532579543 | 13.150703143 | 8.1406664584 | 11.257950951 | 7.644276957 | 7.3262188358 |
| 1986 | 25.672462826 | 13.239252205 | 7.928779213 | 11.295124743 | 7.7971260052 | 6.8349912485 |
| 1987 | 24.685010965 | 13.163921839 | 8.0278835887 | 11.538722742 | 7.7543432779 | 6.43200672 |
| 1988 | 23.676806041 | 13.682024709 | 8.1046324971 | 11.753341372 | 8.1455877263 | 6.3612521368 |
| 1989 | 22.881462192 | 13.936188958 | 8.1166935663 | 11.975382682 | 8.1944207353 | 6.0622482583 |
| 1990 | 21.555821954 | 13.902658473 | 8.3930443919 | 11.563548499 | 8.1913088431 | 5.8416088801 |
| 1991 | 20.930668978 | 13.992423327 | 8.1748721301 | 11.89934746 | 8.6620019251 | 5.713246242 |
| 1992 | 20.143791721 | 14.206569192 | 8.3515351645 | 12.2762067 | 8.7059012682 | 5.6283674023 |
| 1993 | 19.196421444 | 14.00738871 | 8.3664833366 | 12.054824799 | 8.9173345073 | 5.2083196265 |
| 1994 | 18.360738987 | 13.954105365 | 8.3126178364 | 12.299653895 | 9.2661307979 | 5.2470810424 |
| 1995 | 18.53986605 | 14.142926083527676 | 9.1461249441 | 12.475605219 | 9.8794335487 | 5.4110020806 |
| 1996 | 17.605292298 | 14.202032431835278 | 9.1340066151 | 12.625948022 | 9.8556675011 | 5.4271755035 |
| 1997 | 16.767261892 | 14.049129754138068 | 9.0361452877 | 12.462108348 | 10.351526524 | 5.3053454059 |
| 1998 | 16.410294011 | 13.918885565266496 | 9.0485174145 | 12.627914233 | 10.414851658 | 5.237296417 |
| 1999 | 15.93631033 | 13.92504102001458 | 9.1446884404 | 12.461383486 | 10.493153451 | 5.2469635382 |
| 2000 | 15.340630693 | 13.614607394949093 | 8.824630942 | 12.341243646 | 10.697221235 | 5.3097203749 |
| 2001 | 14.59413725 | 13.559061774983986 | 8.8171913038 | 12.215755295 | 11.140424544 | 5.1750473773 |
| 2002 | 13.812854227 | 13.364894013500942 | 8.5184606349 | 11.793023584 | 10.789685822 | 5.2183403094 |
| 2003 | 13.190159419 | 13.52820846838266 | 8.0592999268 | 11.136333321 | 10.898944913 | 5.0922403325 |
| 2004 | 13.153812285 | 13.400942957398234 | 8.0814211719 | 11.54512977 | 11.410950784 | 5.2409859735 |
| 2005 | 12.462227894 | 13.155847007061805 | 7.6881954032 | 11.733144997 | 11.383189574 | 5.1195226381 |
| 2006 | 12.021257844 | 12.651259509295333 | 7.4334151526 | 11.713350072 | 11.695823351 | 5.1212618607 |
| 2007 | 11.524573916 | 12.571103316846017 | 7.27191887 | 11.670059543 | 11.601918435 | 5.0899274006 |
| 2008 | 11.029453169 | 12.319413891475408 | 6.994240157 | 11.700434319 | 11.85833898 | 5.2161053846 |
| 2009 | 10.673839125 | 12.057360009168299 | 6.5706072816 | 11.431400889 | 11.763263001 | 4.996333049 |
| 2010 | 10.231700407 | 12.100346074376569 | 6.3803729444 | 11.529140502 | 11.918322857 | 5.2759591576 |
| 2011 | 9.909214615952374 | 12.0779962685853 | 5.986667468996038 | 11.445367954233006 | 12.054760471927684 | 5.437340594577366 |
| 2012 | 9.565654790280483 | 12.318998255847488 | 5.55625492073955 | 11.411641478258126 | 11.482568683827617 | 5.3706845356157675 |
| 2013 | 9.2 | 12.19473566580682 | 5.2 | 11.40508984986982 | 12.016230934404133 | 5.297914611840531 |
| 2014 | 9.0 | 12.233884507945291 | 5.1 | 11.35475039032296 | 11.840230594359161 | 5.6771516579976495 |
| 2015 | 8.3 | 12.122426819787343 | 4.6 | 11.138839179267459 | 11.955852443257113 | 5.618720513864122 |
| 2016 | 8.0 | 12.029294511113342 | 4.6 | 10.958059183871208 | 12.193521549863124 | 5.469787815147258 |
| 2017 | 7.6 | 11.831408475008184 | 4.0 | 10.332733898468993 | 12.15404580030309 | 5.607113874678353 |
| 2018 | 7.354427876299542 | 11.739280873840922 | 3.7868357492309372 | 10.6 | 12.2 | 5.7 |Stomach
Lung
Stomach
Liver
Uterus
Colorectum
Colorectum
Liver
Lung
Prostate
Breast
“Vital Statistics”, Ministry of Health, Labour and Welfare
(data from e-Stat https://www.e-stat.go.jp/)
