# Supplementary material for: Why has Japan become the world’s most long-lived country: insights from a food and nutrition perspective
Source: Eur J Clin Nutr. 2020 Jul 13;75(6):921–8. doi: 10.1038/s41430-020-0677-5 (PMC8189904; doi:10.1038/s41430-020-0677-5)
Supplement: Supplementary file 3 — Supplemental Figure 3 [file 41430_2020_677_MOESM3_ESM.pptx]

## Slide 1
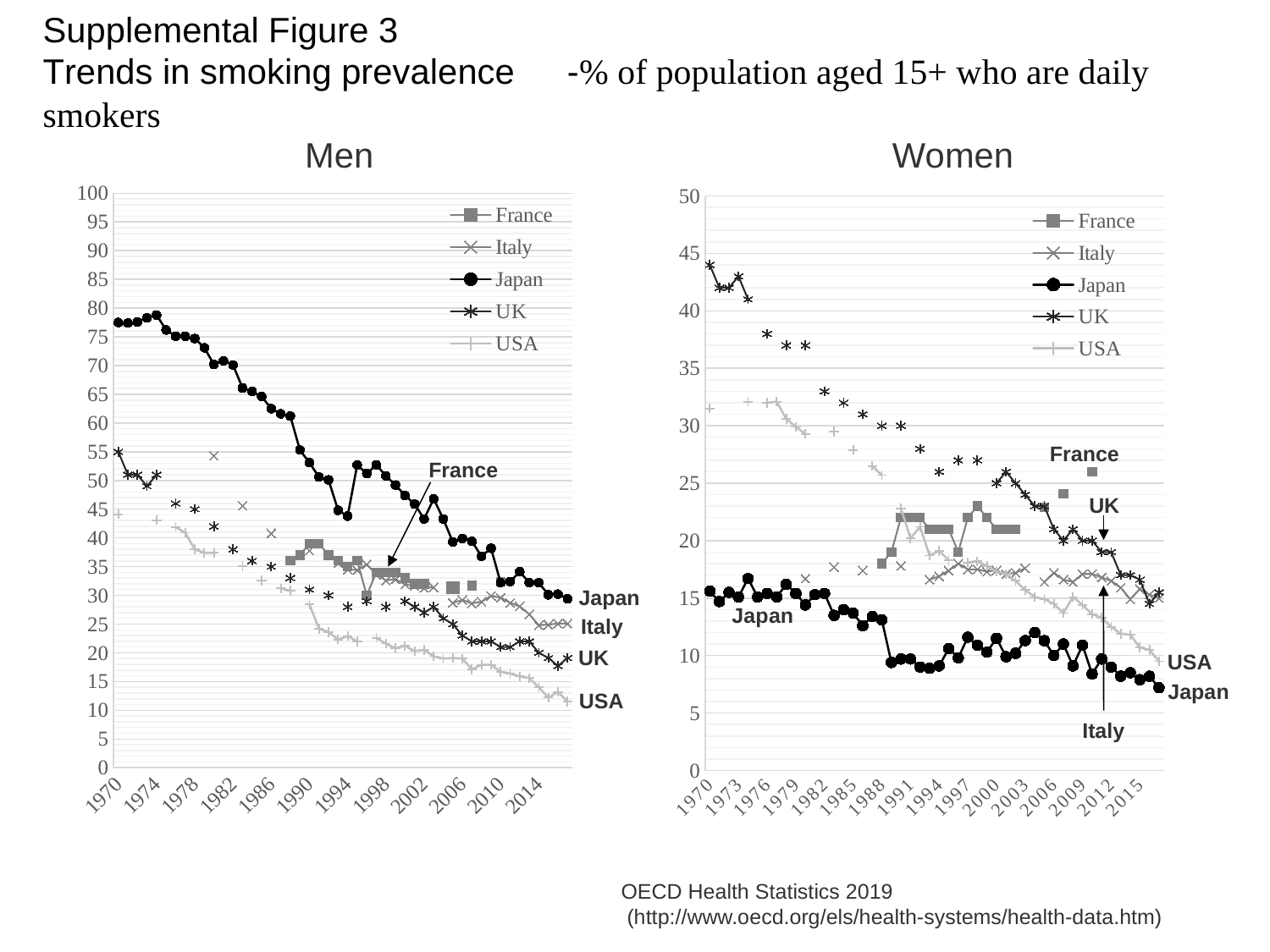

# Supplemental Figure 3 Trends in smoking prevalence　-% of population aged 15+ who are daily smokers
Men
Women
### Chart
| Category | France | Italy | Japan | UK | USA |
|---|---|---|---|---|---|
| 1970 | None | None | 77.5 | 55.0 | 44.1 |
| 1971 | None | None | 77.4 | 51.0 | None |
| 1972 | None | None | 77.6 | 51.0 | None |
| 1973 | None | None | 78.3 | 49.0 | None |
| 1974 | None | None | 78.8 | 51.0 | 43.1 |
| 1975 | None | None | 76.2 | None | None |
| 1976 | None | None | 75.1 | 46.0 | 41.9 |
| 1977 | None | None | 75.1 | None | 40.9 |
| 1978 | None | None | 74.7 | 45.0 | 38.0 |
| 1979 | None | None | 73.1 | None | 37.4 |
| 1980 | None | 54.3 | 70.2 | 42.0 | 37.4 |
| 1981 | None | None | 70.8 | None | None |
| 1982 | None | None | 70.1 | 38.0 | None |
| 1983 | None | 45.6 | 66.1 | None | 35.1 |
| 1984 | None | None | 65.5 | 36.0 | None |
| 1985 | None | None | 64.6 | None | 32.6 |
| 1986 | None | 40.8 | 62.5 | 35.0 | None |
| 1987 | None | None | 61.6 | None | 31.2 |
| 1988 | 36.0 | None | 61.2 | 33.0 | 30.8 |
| 1989 | 37.0 | None | 55.3 | None | None |
| 1990 | 39.0 | 37.8 | 53.1 | 31.0 | 28.4 |
| 1991 | 39.0 | None | 50.6 | None | 24.2 |
| 1992 | 37.0 | None | 50.1 | 30.0 | 23.6 |
| 1993 | 36.0 | 35.6 | 44.8 | None | 22.3 |
| 1994 | 35.0 | 34.5 | 43.8 | 28.0 | 22.9 |
| 1995 | 36.0 | 34.4 | 52.7 | None | 22.0 |
| 1996 | 30.0 | 35.4 | 51.2 | 29.0 | None |
| 1997 | 34.0 | 33.6 | 52.7 | None | 22.6 |
| 1998 | 34.0 | 32.6 | 50.8 | 28.0 | 21.6 |
| 1999 | 34.0 | 32.8 | 49.2 | None | 20.8 |
| 2000 | 33.0 | 31.9 | 47.4 | 29.0 | 21.2 |
| 2001 | 32.0 | 31.6 | 45.9 | 28.0 | 20.3 |
| 2002 | 32.0 | 31.3 | 43.3 | 27.0 | 20.5 |
| 2003 | None | 31.4 | 46.8 | 28.0 | 19.4 |
| 2004 | None | None | 43.3 | 26.0 | 19.0 |
| 2005 | 31.3 | 28.7 | 39.3 | 25.0 | 19.1 |
| 2006 | None | 29.2 | 39.9 | 23.0 | 19.0 |
| 2007 | 31.7 | 28.6 | 39.4 | 22.0 | 17.1 |
| 2008 | None | 28.9 | 36.8 | 22.0 | 17.9 |
| 2009 | None | 29.9 | 38.2 | 22.0 | 17.9 |
| 2010 | 32.4 | 29.6 | 32.2 | 21.0 | 16.7 |
| 2011 | None | 28.7 | 32.4 | 21.0 | 16.4 |
| 2012 | None | 28.1 | 34.1 | 22.0 | 15.9 |
| 2013 | None | 26.7 | 32.2 | 22.0 | 15.6 |
| 2014 | None | 24.8 | 32.2 | 20.0 | 14.0 |
| 2015 | None | 24.9 | 30.1 | 19.1 | 12.2 |
| 2016 | None | 25.1 | 30.2 | 17.7 | 13.2 |
| 2017 | None | 25.1 | 29.4 | 19.1 | 11.5 |
### Chart
| Category | France | Italy | Japan | UK | USA |
|---|---|---|---|---|---|
| 1970 | None | None | 15.6 | 44.0 | 31.5 |
| 1971 | None | None | 14.7 | 42.0 | None |
| 1972 | None | None | 15.5 | 42.0 | None |
| 1973 | None | None | 15.1 | 43.0 | None |
| 1974 | None | None | 16.7 | 41.0 | 32.1 |
| 1975 | None | None | 15.1 | None | None |
| 1976 | None | None | 15.4 | 38.0 | 32.0 |
| 1977 | None | None | 15.1 | None | 32.1 |
| 1978 | None | None | 16.2 | 37.0 | 30.6 |
| 1979 | None | None | 15.4 | None | 29.9 |
| 1980 | None | 16.7 | 14.4 | 37.0 | 29.3 |
| 1981 | None | None | 15.3 | None | None |
| 1982 | None | None | 15.4 | 33.0 | None |
| 1983 | None | 17.7 | 13.5 | None | 29.5 |
| 1984 | None | None | 14.0 | 32.0 | None |
| 1985 | None | None | 13.7 | None | 27.9 |
| 1986 | None | 17.4 | 12.6 | 31.0 | None |
| 1987 | None | None | 13.4 | None | 26.5 |
| 1988 | 18.0 | None | 13.1 | 30.0 | 25.7 |
| 1989 | 19.0 | None | 9.4 | None | None |
| 1990 | 22.0 | 17.8 | 9.7 | 30.0 | 22.8 |
| 1991 | 22.0 | None | 9.7 | None | 20.2 |
| 1992 | 22.0 | None | 9.0 | 28.0 | 21.2 |
| 1993 | 21.0 | 16.6 | 8.9 | None | 18.7 |
| 1994 | 21.0 | 16.9 | 9.1 | 26.0 | 19.1 |
| 1995 | 21.0 | 17.4 | 10.6 | None | 18.3 |
| 1996 | 19.0 | 18.0 | 9.8 | 27.0 | None |
| 1997 | 22.0 | 17.5 | 11.6 | None | 18.1 |
| 1998 | 23.0 | 17.5 | 10.9 | 27.0 | 18.2 |
| 1999 | 22.0 | 17.3 | 10.3 | None | 17.8 |
| 2000 | 21.0 | 17.4 | 11.5 | 25.0 | 17.3 |
| 2001 | 21.0 | 17.1 | 9.9 | 26.0 | 17.1 |
| 2002 | 21.0 | 17.2 | 10.2 | 25.0 | 16.5 |
| 2003 | None | 17.6 | 11.3 | 24.0 | 15.7 |
| 2004 | None | None | 12.0 | 23.0 | 15.1 |
| 2005 | 22.9 | 16.4 | 11.3 | 23.0 | 14.9 |
| 2006 | None | 17.2 | 10.0 | 21.0 | 14.5 |
| 2007 | 24.1 | 16.6 | 11.0 | 20.0 | 13.7 |
| 2008 | None | 16.4 | 9.1 | 21.0 | 15.1 |
| 2009 | None | 17.1 | 10.9 | 20.0 | 14.4 |
| 2010 | 26.0 | 17.1 | 8.4 | 20.0 | 13.6 |
| 2011 | None | 16.8 | 9.7 | 19.0 | 13.3 |
| 2012 | None | 16.5 | 9.0 | 19.0 | 12.5 |
| 2013 | None | 15.9 | 8.2 | 17.0 | 11.9 |
| 2014 | None | 14.9 | 8.5 | 17.0 | 11.8 |
| 2015 | None | 15.8 | 7.9 | 16.6 | 10.7 |
| 2016 | None | 15.3 | 8.2 | 14.5 | 10.5 |
| 2017 | None | 15.0 | 7.2 | 15.5 | 9.5 |France
France
UK
Japan
Italy
UK
USA
Japan
USA
Italy
OECD Health Statistics 2019
 (http://www.oecd.org/els/health-systems/health-data.htm)
