# Supplementary material for: Why has Japan become the world’s most long-lived country: insights from a food and nutrition perspective
Source: Eur J Clin Nutr. 2020 Jul 13;75(6):921–8. doi: 10.1038/s41430-020-0677-5 (PMC8189904; doi:10.1038/s41430-020-0677-5)
Supplement: Supplementary file 4 — Supplemental Figure 4 [file 41430_2020_677_MOESM4_ESM.pptx]

## Slide 1
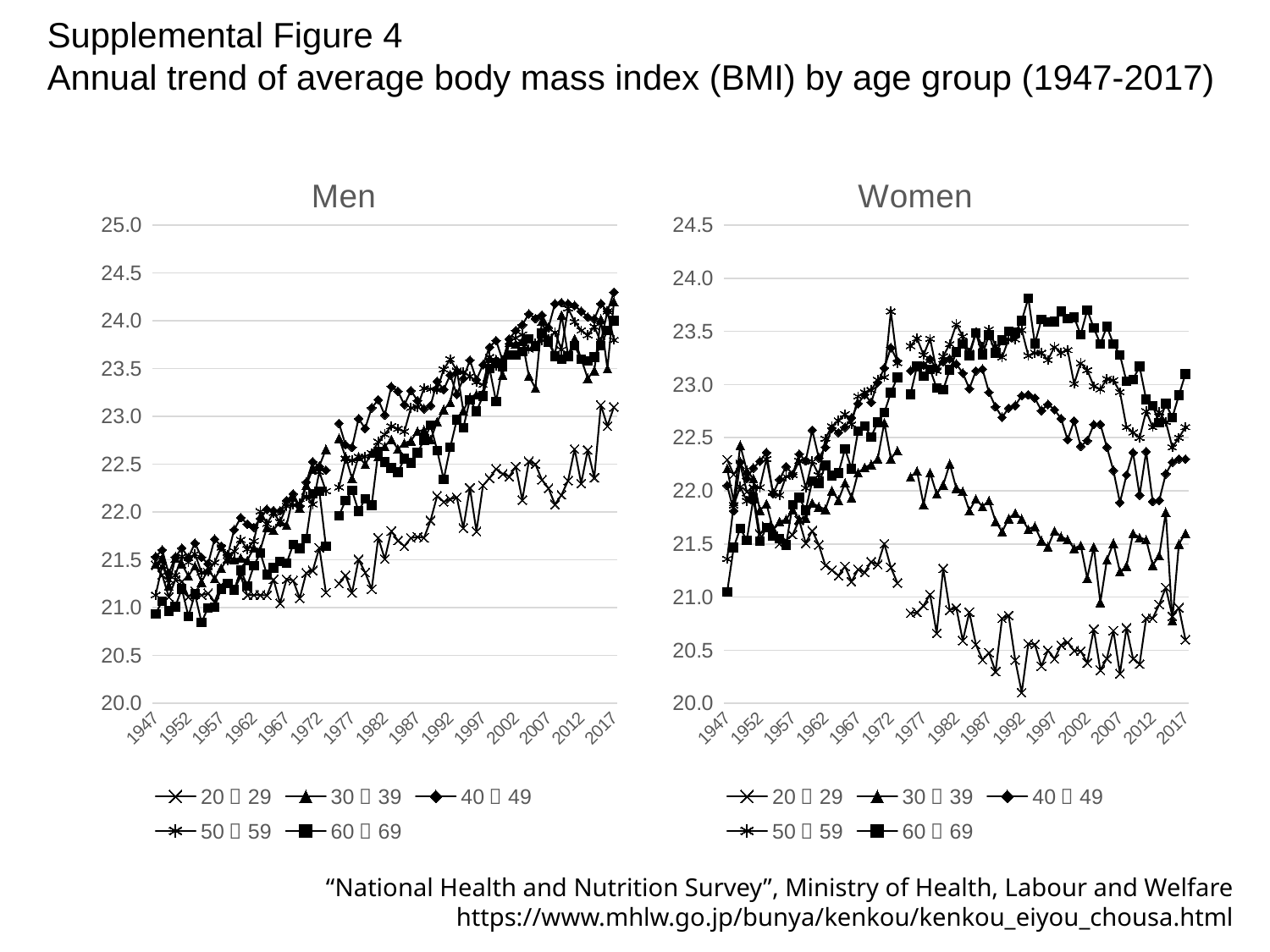

Supplemental Figure 4
Annual trend of average body mass index (BMI) by age group (1947-2017)
### Chart: Men
| Category | 20－29 | 30－39 | 40－49 | 50－59 | 60－69 |
|---|---|---|---|---|---|
| 1947 | 21.44390524130976 | 21.4575446872875 | 21.532068408687635 | 21.133135545260167 | 20.936452970836832 |
| 1948 | 21.34317074267546 | 21.515625 | 21.604096212779726 | 21.43714005705807 | 21.068121633179036 |
| 1949 | 21.11397597983303 | 21.232802344636866 | 21.356775482453354 | 21.29302091206853 | 20.967318126149586 |
| 1950 | 21.28911667099905 | 21.516892258705507 | 21.53060051412704 | 21.340645536455796 | 21.011843374764698 |
| 1951 | 21.277503360116878 | 21.455537116293517 | 21.622375671339885 | 21.537426046671516 | 21.19704214388969 |
| 1952 | 21.11419854128604 | 21.331314719383546 | 21.525959169686118 | 21.477187114972825 | 20.910871854579703 |
| 1953 | 21.176531292932715 | 21.436580444751858 | 21.675248002431072 | 21.564503317185597 | 21.14230675870221 |
| 1954 | 21.13177611079231 | 21.268524449474814 | 21.527234160753522 | 21.368757037915184 | 20.847410915697825 |
| 1955 | 21.15258597490425 | 21.398292860768986 | 21.458721992952306 | 21.37975428485242 | 20.999038465080805 |
| 1956 | 21.060205244500075 | 21.305215055992406 | 21.71782840560047 | 21.472510778938357 | 21.008369094862246 |
| 1957 | 21.2398713078944 | 21.41270082564426 | 21.64423433814364 | 21.631331124936423 | 21.195943818923517 |
| 1958 | 21.2335897786219 | 21.51250904032098 | 21.550367040688734 | 21.49488998795888 | 21.253078588863826 |
| 1959 | 21.19237059529267 | 21.50483148534585 | 21.81632438347763 | 21.593778696051423 | 21.188084464738854 |
| 1960 | 21.32853431855352 | 21.521109586953205 | 21.941480780084174 | 21.71074129793546 | 21.39200292758524 |
| 1961 | 21.131073027263394 | 21.49395205318874 | 21.874965905601773 | 21.61306910327914 | 21.228690376758674 |
| 1962 | 21.132713440405745 | 21.62733804459816 | 21.839850156733725 | 21.69724812217923 | 21.4399366957231 |
| 1963 | 21.132713440405745 | 21.608598849765826 | 21.9330169544162 | 22.008609378273615 | 21.573525590170103 |
| 1964 | 21.129786277225026 | 21.842930606427835 | 22.02847112379924 | 21.87809026541672 | 21.344864206064646 |
| 1965 | 21.296888218280923 | 21.812310620298447 | 22.01689749623857 | 21.99276143757409 | 21.415971264157523 |
| 1966 | 21.046831955922862 | 21.90198297358233 | 22.01194648717853 | 21.89428088754591 | 21.482489292013103 |
| 1967 | 21.298784921946783 | 21.864756201365534 | 22.11956557250086 | 22.06312791399341 | 21.466035573777265 |
| 1968 | 21.284946285630834 | 22.162003074266636 | 22.190685962160572 | 22.075081186675966 | 21.66039882943545 |
| 1969 | 21.098857605992656 | 22.04377855325298 | 22.057224871944186 | 22.093468284383476 | 21.62044236599801 |
| 1970 | 21.364097633926182 | 22.280204621758365 | 22.31367169638775 | 22.164415611539575 | 21.722729571669966 |
| 1971 | 21.389876331592554 | 22.452301999231068 | 22.52657964293268 | 22.0817641690995 | 22.1915696377135 |
| 1972 | 21.62659581764652 | 22.48220782014234 | 22.477882070385256 | 22.422328698728624 | 22.21986618522269 |
| 1973 | 21.158848693236596 | 22.656130620346797 | 22.4395892559553 | 22.22033781194842 | 21.641605448423626 |
| 1974 | None | None | None | None | None |
| 1975 | 21.25410470354361 | 22.772641820260866 | 22.928770876722183 | 22.25994367501254 | 21.963856853263433 |
| 1976 | 21.33972015034089 | 22.579244463742945 | 22.703842540352216 | 22.558008653159416 | 22.119872972145696 |
| 1977 | 21.15677831310906 | 22.351658273191497 | 22.675959408146134 | 22.54056434637565 | 22.22529593336194 |
| 1978 | 21.505635761963955 | 22.580806899699127 | 22.977802461831942 | 22.579095225600224 | 22.008578190331765 |
| 1979 | 21.367875108993015 | 22.49963710262738 | 22.87480842347945 | 22.582407576033507 | 22.139306536719367 |
| 1980 | 21.192735348402163 | 22.61753779829143 | 23.09057814302192 | 22.617626104824797 | 22.07191171235315 |
| 1981 | 21.731425475667947 | 22.581291744127505 | 23.17480591100049 | 22.735768578247658 | 22.630346862213855 |
| 1982 | 21.509844478180444 | 22.688427932470336 | 23.0145746579417 | 22.814317777503938 | 22.523363567575004 |
| 1983 | 21.80249542346181 | 22.760226755041444 | 23.312016656751936 | 22.899103029114794 | 22.4609375 |
| 1984 | 21.7019711606815 | 22.6612642977518 | 23.264006839914362 | 22.87222645711496 | 22.415861712813115 |
| 1985 | 21.643458386027408 | 22.72138145999277 | 23.12305589593741 | 22.84403265214278 | 22.560750372895306 |
| 1986 | 21.72946846244529 | 22.746598639455783 | 23.270336506739557 | 23.090993606964123 | 22.515309439931677 |
| 1987 | 21.740439108710795 | 22.844648233211228 | 23.166370773921468 | 23.10059171597633 | 22.62112797805984 |
| 1988 | 21.730707390808515 | 22.858350430134987 | 23.080272898824212 | 23.29626405828411 | 22.753749468150318 |
| 1989 | 21.91269775781744 | 22.758306781975424 | 23.113905325443785 | 23.287304968820884 | 22.907491018610795 |
| 1990 | 22.169988904709886 | 22.944733410659076 | 23.36671366494083 | 23.27964551551775 | 22.645731260368045 |
| 1991 | 22.106766299262386 | 23.073421798956623 | 23.282685994294848 | 23.49222381206221 | 22.343132429720786 |
| 1992 | 22.136508231800814 | 23.1463353086033 | 23.43448981821737 | 23.598931085099178 | 22.67687869292008 |
| 1993 | 22.154588747396712 | 23.487832228977954 | 23.231116421080575 | 23.477800311850466 | 22.965810081568883 |
| 1994 | 21.832403057157183 | 23.059896354741294 | 23.39697809182354 | 23.46615422991288 | 22.882042689833533 |
| 1995 | 22.253506631238263 | 23.197979566446335 | 23.589582414728977 | 23.420711456834848 | 23.17653873191663 |
| 1996 | 21.798278430736055 | 23.232500369372595 | 23.3843537414966 | 23.360881542699723 | 23.05321653036958 |
| 1997 | 22.27951131103153 | 23.246875619916683 | 23.540855756057354 | 23.332591043880313 | 23.214201183431953 |
| 1998 | 22.355375746377813 | 23.61138137219877 | 23.724717024496826 | 23.62154416261261 | 23.49922877733019 |
| 1999 | 22.452544576013686 | 23.597004206422486 | 23.79416442884062 | 23.528134878128995 | 23.157163855633787 |
| 2000 | 22.400054717690914 | 23.43291520583171 | 23.58437591249073 | 23.585034820784767 | 23.52365538785803 |
| 2001 | 22.371630868090676 | 23.77438112314139 | 23.808690171912748 | 23.78500957916825 | 23.64518538539585 |
| 2002 | 22.476354567567576 | 23.761581631133286 | 23.898830841284198 | 23.821212029060433 | 23.64518538539585 |
| 2003 | 22.1264662631237 | 23.789413642371755 | 23.957437230824045 | 23.857414478952624 | 23.67926381056589 |
| 2004 | 22.530733275419745 | 23.42482422931314 | 24.07343119751372 | 23.702383604366226 | 23.811607899222754 |
| 2005 | 22.50265038815362 | 23.296439380355462 | 24.022950783196865 | 23.76639560128981 | 23.737428435362244 |
| 2006 | 22.33763142908142 | 23.9975818606874 | 24.06099963960282 | 23.794815802758833 | 23.869720404521118 |
| 2007 | 22.25 | 23.79 | 23.93 | 23.81 | 23.78 |
| 2008 | 22.08 | 23.65 | 24.18 | 23.88 | 23.63 |
| 2009 | 22.18 | 24.06 | 24.19 | 23.7 | 23.6 |
| 2010 | 22.33 | 23.64 | 24.18 | 24.13 | 23.63 |
| 2011 | 22.66 | 23.81 | 24.16 | 23.99 | 23.74 |
| 2012 | 22.3 | 23.6 | 24.1 | 23.9 | 23.6 |
| 2013 | 22.65 | 23.4 | 24.04 | 23.85 | 23.58 |
| 2014 | 22.36 | 23.48 | 24.02 | 23.94 | 23.62 |
| 2015 | 23.12 | 24.02 | 24.18 | 23.78 | 23.74 |
| 2016 | 22.9 | 23.5 | 24.1 | 24.1 | 23.9 |
| 2017 | 23.1 | 24.2 | 24.3 | 23.8 | 24.0 |
### Chart: Women
| Category | 20－29 | 30－39 | 40－49 | 50－59 | 60－69 |
|---|---|---|---|---|---|
| 1947 | 22.293360897068837 | 22.209971286681387 | 22.05099726965616 | 21.36016343689848 | 21.04710568208546 |
| 1948 | 22.154282324942997 | 21.900111284541566 | 21.81181179338291 | 21.863867588455506 | 21.46663649698824 |
| 1949 | 22.248492931780103 | 22.430074141969286 | 22.27006229810266 | 22.040363245592285 | 21.646086246383593 |
| 1950 | 21.994084380425956 | 22.190940413265672 | 22.11751482561085 | 21.903734284105838 | 21.534506471474632 |
| 1951 | 21.974071163100344 | 22.114264399642597 | 22.214755851998106 | 22.03368403574448 | 21.92853236613393 |
| 1952 | 21.592874151644647 | 21.81631077952148 | 22.279035792549305 | 22.033046290010734 | 21.526725023228522 |
| 1953 | 21.6679231821512 | 21.877178733405692 | 22.359460209441075 | 22.300194345700454 | 21.653777513384888 |
| 1954 | 21.630440732899196 | 21.64151618021856 | 21.973152469873888 | 21.981795676850624 | 21.57607837900181 |
| 1955 | 21.50235781943914 | 21.708809451923216 | 22.107623870397994 | 21.961053282640453 | 21.55268834773745 |
| 1956 | 21.53519455921188 | 21.731282649913503 | 22.22902424922627 | 22.136630966470257 | 21.49184806164161 |
| 1957 | 21.587719423990446 | 21.820528163876183 | 22.15646929451643 | 22.15016858583261 | 21.869577703103534 |
| 1958 | 21.722166232748243 | 21.733333333333334 | 22.346926050629754 | 22.28343635223506 | 21.93737533105824 |
| 1959 | 21.507556739852255 | 21.742222222222225 | 22.28344046525865 | 22.027858762552643 | 21.817129629629633 |
| 1960 | 21.62480248316484 | 21.885702978606986 | 22.572276943575154 | 22.277754639270672 | 22.091498182974274 |
| 1961 | 21.49172469273296 | 21.850289219741928 | 22.311105330095963 | 22.148178999490952 | 22.072288184554292 |
| 1962 | 21.297427193193098 | 21.826188879534804 | 22.41003281309083 | 22.491756082872488 | 22.23905694920313 |
| 1963 | 21.254314992397163 | 22.001852371523015 | 22.59035133835192 | 22.608064893246848 | 22.14340509135709 |
| 1964 | 21.201104684630355 | 21.912986652919404 | 22.54787910267206 | 22.661816291343293 | 22.16927479316579 |
| 1965 | 21.288750111456636 | 22.07504551883146 | 22.599010859363215 | 22.722595023948806 | 22.392288086444246 |
| 1966 | 21.14571318723568 | 21.936847150061542 | 22.68620964931422 | 22.626332265122308 | 22.208981611268044 |
| 1967 | 21.26096708001282 | 22.1721182019192 | 22.82152967444162 | 22.889760226675826 | 22.56315977202529 |
| 1968 | 21.233238400758438 | 22.220043786556875 | 22.906086824171414 | 22.928282211637757 | 22.6105912619812 |
| 1969 | 21.33305229232571 | 22.24840078755863 | 22.83418505314511 | 22.948325572491832 | 22.508430003850872 |
| 1970 | 21.306782151680615 | 22.302535233975092 | 23.01991679144423 | 23.04455067894014 | 22.646910095399107 |
| 1971 | 21.50303677274265 | 22.642042827053185 | 23.155555555555555 | 23.069617814851277 | 22.734839476813317 |
| 1972 | 21.279486820221567 | 22.30443741595312 | 23.346638550093857 | 23.6894741891584 | 22.92499439793938 |
| 1973 | 21.133145634642002 | 22.377077562326875 | 23.222701736183925 | 23.20347861465697 | 23.06673409008574 |
| 1974 | None | None | None | None | None |
| 1975 | 20.847875110741228 | 22.129501327126786 | 23.13039562650831 | 23.363632386324422 | 22.909121062758345 |
| 1976 | 20.857440166493234 | 22.186206518461663 | 23.170037856075083 | 23.435943179758183 | 23.171887010528785 |
| 1977 | 20.919776802634484 | 21.871512100180034 | 23.18314156938496 | 23.27954759930224 | 23.07882722333791 |
| 1978 | 21.025567089580935 | 22.170959887222864 | 23.239874382363205 | 23.43152171380748 | 23.14192719997611 |
| 1979 | 20.659702435308496 | 21.97049409388923 | 23.14798699535648 | 23.13173230724489 | 22.968415886379827 |
| 1980 | 21.270341059345903 | 22.055486334097694 | 23.217221189381355 | 23.265958993747272 | 22.952175133392227 |
| 1981 | 20.877289503722093 | 22.254476319832992 | 23.242728531855956 | 23.38232465822315 | 23.13850710352168 |
| 1982 | 20.89938133220499 | 22.02401080397746 | 23.194364028214345 | 23.568564772052923 | 23.306430174858225 |
| 1983 | 20.589545917469103 | 21.995454553101155 | 23.108139626622467 | 23.453483109264305 | 23.381304897739007 |
| 1984 | 20.858578166777015 | 21.812666111841924 | 22.961569470572428 | 23.29780160706202 | 23.27208231431908 |
| 1985 | 20.554391754520445 | 21.923622117085532 | 23.12847783777857 | 23.492866147400452 | 23.483806702177716 |
| 1986 | 20.40957366623436 | 21.852237252861602 | 23.146199814830403 | 23.355150605401708 | 23.283418553688822 |
| 1987 | 20.476573098067234 | 21.907207388091376 | 22.92713661878631 | 23.51884336803984 | 23.46603360116874 |
| 1988 | 20.299254203711296 | 21.712921139334682 | 22.790692739445515 | 23.36324443443163 | 23.294294577923726 |
| 1989 | 20.799746194536713 | 21.614941369471538 | 22.692928135661212 | 23.257637243054575 | 23.416658747440646 |
| 1990 | 20.827648784488726 | 21.738691033800578 | 22.77792037586968 | 23.427315988685088 | 23.503348434649826 |
| 1991 | 20.407288913644905 | 21.791579750000107 | 22.80341254340278 | 23.427315988685088 | 23.48966658730968 |
| 1992 | 20.101380835254957 | 21.735846832276625 | 22.89593523359757 | 23.514568562199738 | 23.60254042610693 |
| 1993 | 20.561555353152745 | 21.639498208617013 | 22.903299417434024 | 23.273341276029196 | 23.814667760577795 |
| 1994 | 20.555472231012626 | 21.665745600978056 | 22.87366072831244 | 23.298627455811495 | 23.386719569039208 |
| 1995 | 20.34930299631469 | 21.530316153119696 | 22.755679438326478 | 23.298627455811495 | 23.610730292880582 |
| 1996 | 20.499422237962552 | 21.4745216955472 | 22.81455568652801 | 23.23208552842903 | 23.586994398311347 |
| 1997 | 20.417752816531113 | 21.62359527769889 | 22.76299657228246 | 23.35488170495769 | 23.592324630386912 |
| 1998 | 20.548746698159018 | 21.568608407953434 | 22.68007126600821 | 23.299799895836184 | 23.688888888888886 |
| 1999 | 20.575628268586946 | 21.53978458604963 | 22.48118901977479 | 23.324362424708443 | 23.620048343837265 |
| 2000 | 20.49541139424548 | 21.45926202684944 | 22.658324009089693 | 23.007860166747342 | 23.632781842968985 |
| 2001 | 20.492991316447217 | 21.486520534139583 | 22.419466162590727 | 23.202881576912027 | 23.469330692556614 |
| 2002 | 20.380340136731384 | 21.17725482614797 | 22.47265519947244 | 23.13568161211816 | 23.702059396745202 |
| 2003 | 20.697448060593178 | 21.472265593428684 | 22.62439729753214 | 22.985401518560394 | 23.531749097692078 |
| 2004 | 20.31215269311609 | 20.950168242268866 | 22.626079461003727 | 22.957447031346554 | 23.38368970192776 |
| 2005 | 20.422374356794347 | 21.349708626359746 | 22.41090951686797 | 23.053274401744524 | 23.54566220318377 |
| 2006 | 20.683632045787242 | 21.507547916908514 | 22.1915696377135 | 23.04123333438066 | 23.38368970192776 |
| 2007 | 20.28 | 21.24 | 21.89 | 22.93 | 23.28 |
| 2008 | 20.71 | 21.29 | 22.15 | 22.6 | 23.03 |
| 2009 | 20.42 | 21.6 | 22.36 | 22.55 | 23.05 |
| 2010 | 20.37 | 21.56 | 21.96 | 22.5 | 23.17 |
| 2011 | 20.8 | 21.54 | 22.37 | 22.75 | 22.86 |
| 2012 | 20.8 | 21.3 | 21.9 | 22.6 | 22.8 |
| 2013 | 20.93 | 21.39 | 21.91 | 22.74 | 22.64 |
| 2014 | 21.09 | 21.8 | 22.16 | 22.65 | 22.82 |
| 2015 | 20.82 | 20.78 | 22.27 | 22.41 | 22.69 |
| 2016 | 20.9 | 21.5 | 22.3 | 22.5 | 22.9 |
| 2017 | 20.6 | 21.6 | 22.3 | 22.6 | 23.1 |“National Health and Nutrition Survey”, Ministry of Health, Labour and Welfare
https://www.mhlw.go.jp/bunya/kenkou/kenkou_eiyou_chousa.html
