# Supplementary material for: Why has Japan become the world’s most long-lived country: insights from a food and nutrition perspective
Source: Eur J Clin Nutr. 2020 Jul 13;75(6):921–8. doi: 10.1038/s41430-020-0677-5 (PMC8189904; doi:10.1038/s41430-020-0677-5)
Supplement: Supplementary file 5 — Supplemental Figure 5 [file 41430_2020_677_MOESM5_ESM.pptx]

## Slide 1
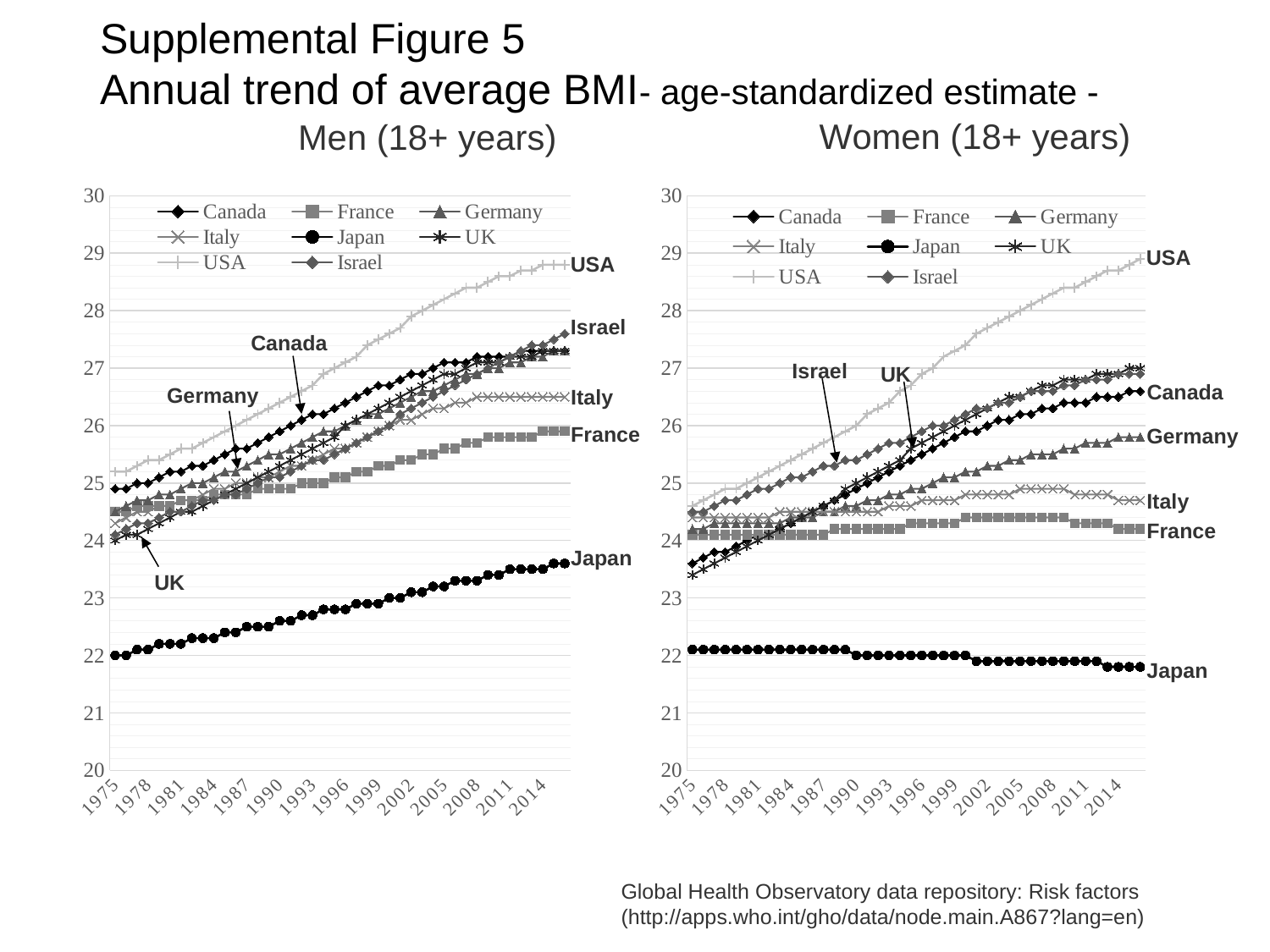

# Supplemental Figure 5 Annual trend of average BMI- age-standardized estimate -
Women (18+ years)
Men (18+ years)
### Chart
| Category | Canada | France | Germany | Italy | Japan | UK | USA | Israel |
|---|---|---|---|---|---|---|---|---|
| 1975 | 24.9 | 24.5 | 24.5 | 24.3 | 22.0 | 24.0 | 25.2 | 24.1 |
| 1976 | 24.9 | 24.5 | 24.6 | 24.4 | 22.0 | 24.1 | 25.2 | 24.2 |
| 1977 | 25.0 | 24.6 | 24.7 | 24.5 | 22.1 | 24.1 | 25.3 | 24.3 |
| 1978 | 25.0 | 24.6 | 24.7 | 24.5 | 22.1 | 24.2 | 25.4 | 24.3 |
| 1979 | 25.1 | 24.6 | 24.8 | 24.6 | 22.2 | 24.3 | 25.4 | 24.4 |
| 1980 | 25.2 | 24.6 | 24.8 | 24.6 | 22.2 | 24.4 | 25.5 | 24.5 |
| 1981 | 25.2 | 24.7 | 24.9 | 24.7 | 22.2 | 24.5 | 25.6 | 24.5 |
| 1982 | 25.3 | 24.7 | 25.0 | 24.7 | 22.3 | 24.5 | 25.6 | 24.6 |
| 1983 | 25.3 | 24.7 | 25.0 | 24.8 | 22.3 | 24.6 | 25.7 | 24.7 |
| 1984 | 25.4 | 24.8 | 25.1 | 24.9 | 22.3 | 24.7 | 25.8 | 24.7 |
| 1985 | 25.5 | 24.8 | 25.2 | 24.9 | 22.4 | 24.8 | 25.9 | 24.8 |
| 1986 | 25.6 | 24.8 | 25.2 | 25.0 | 22.4 | 24.9 | 26.0 | 24.8 |
| 1987 | 25.6 | 24.8 | 25.3 | 25.0 | 22.5 | 25.0 | 26.1 | 24.9 |
| 1988 | 25.7 | 24.9 | 25.4 | 25.1 | 22.5 | 25.1 | 26.2 | 25.0 |
| 1989 | 25.8 | 24.9 | 25.5 | 25.1 | 22.5 | 25.2 | 26.3 | 25.1 |
| 1990 | 25.9 | 24.9 | 25.5 | 25.2 | 22.6 | 25.3 | 26.4 | 25.1 |
| 1991 | 26.0 | 24.9 | 25.6 | 25.3 | 22.6 | 25.4 | 26.5 | 25.2 |
| 1992 | 26.1 | 25.0 | 25.7 | 25.3 | 22.7 | 25.5 | 26.6 | 25.3 |
| 1993 | 26.2 | 25.0 | 25.8 | 25.4 | 22.7 | 25.6 | 26.7 | 25.4 |
| 1994 | 26.2 | 25.0 | 25.9 | 25.5 | 22.8 | 25.7 | 26.9 | 25.4 |
| 1995 | 26.3 | 25.1 | 25.9 | 25.6 | 22.8 | 25.8 | 27.0 | 25.5 |
| 1996 | 26.4 | 25.1 | 26.0 | 25.6 | 22.8 | 26.0 | 27.1 | 25.6 |
| 1997 | 26.5 | 25.2 | 26.1 | 25.7 | 22.9 | 26.1 | 27.2 | 25.7 |
| 1998 | 26.6 | 25.2 | 26.2 | 25.8 | 22.9 | 26.2 | 27.4 | 25.8 |
| 1999 | 26.7 | 25.3 | 26.2 | 25.9 | 22.9 | 26.3 | 27.5 | 25.9 |
| 2000 | 26.7 | 25.3 | 26.3 | 26.0 | 23.0 | 26.4 | 27.6 | 26.0 |
| 2001 | 26.8 | 25.4 | 26.4 | 26.1 | 23.0 | 26.5 | 27.7 | 26.2 |
| 2002 | 26.9 | 25.4 | 26.5 | 26.1 | 23.1 | 26.6 | 27.9 | 26.3 |
| 2003 | 26.9 | 25.5 | 26.6 | 26.2 | 23.1 | 26.7 | 28.0 | 26.4 |
| 2004 | 27.0 | 25.5 | 26.6 | 26.3 | 23.2 | 26.8 | 28.1 | 26.5 |
| 2005 | 27.1 | 25.6 | 26.7 | 26.3 | 23.2 | 26.9 | 28.2 | 26.6 |
| 2006 | 27.1 | 25.6 | 26.8 | 26.4 | 23.3 | 26.9 | 28.3 | 26.7 |
| 2007 | 27.1 | 25.7 | 26.9 | 26.4 | 23.3 | 27.0 | 28.4 | 26.8 |
| 2008 | 27.2 | 25.7 | 26.9 | 26.5 | 23.3 | 27.1 | 28.4 | 26.9 |
| 2009 | 27.2 | 25.8 | 27.0 | 26.5 | 23.4 | 27.1 | 28.5 | 27.0 |
| 2010 | 27.2 | 25.8 | 27.0 | 26.5 | 23.4 | 27.1 | 28.6 | 27.1 |
| 2011 | 27.2 | 25.8 | 27.1 | 26.5 | 23.5 | 27.2 | 28.6 | 27.2 |
| 2012 | 27.3 | 25.8 | 27.1 | 26.5 | 23.5 | 27.2 | 28.7 | 27.3 |
| 2013 | 27.3 | 25.8 | 27.2 | 26.5 | 23.5 | 27.2 | 28.7 | 27.4 |
| 2014 | 27.3 | 25.9 | 27.2 | 26.5 | 23.5 | 27.3 | 28.8 | 27.4 |
| 2015 | 27.3 | 25.9 | 27.3 | 26.5 | 23.6 | 27.3 | 28.8 | 27.5 |
| 2016 | 27.3 | 25.9 | 27.3 | 26.5 | 23.6 | 27.3 | 28.8 | 27.6 |
### Chart
| Category | Canada | France | Germany | Italy | Japan | UK | USA | Israel |
|---|---|---|---|---|---|---|---|---|
| 1975 | 23.6 | 24.1 | 24.2 | 24.4 | 22.1 | 23.4 | 24.6 | 24.5 |
| 1976 | 23.7 | 24.1 | 24.2 | 24.4 | 22.1 | 23.5 | 24.7 | 24.5 |
| 1977 | 23.8 | 24.1 | 24.3 | 24.4 | 22.1 | 23.6 | 24.8 | 24.6 |
| 1978 | 23.8 | 24.1 | 24.3 | 24.4 | 22.1 | 23.7 | 24.9 | 24.7 |
| 1979 | 23.9 | 24.1 | 24.3 | 24.4 | 22.1 | 23.8 | 24.9 | 24.7 |
| 1980 | 24.0 | 24.1 | 24.3 | 24.4 | 22.1 | 23.9 | 25.0 | 24.8 |
| 1981 | 24.1 | 24.1 | 24.3 | 24.4 | 22.1 | 24.0 | 25.1 | 24.9 |
| 1982 | 24.1 | 24.1 | 24.3 | 24.4 | 22.1 | 24.1 | 25.2 | 24.9 |
| 1983 | 24.2 | 24.1 | 24.3 | 24.5 | 22.1 | 24.2 | 25.3 | 25.0 |
| 1984 | 24.3 | 24.1 | 24.4 | 24.5 | 22.1 | 24.3 | 25.4 | 25.1 |
| 1985 | 24.4 | 24.1 | 24.4 | 24.5 | 22.1 | 24.4 | 25.5 | 25.1 |
| 1986 | 24.5 | 24.1 | 24.4 | 24.5 | 22.1 | 24.5 | 25.6 | 25.2 |
| 1987 | 24.6 | 24.1 | 24.5 | 24.5 | 22.1 | 24.6 | 25.7 | 25.3 |
| 1988 | 24.7 | 24.2 | 24.5 | 24.5 | 22.1 | 24.7 | 25.8 | 25.3 |
| 1989 | 24.8 | 24.2 | 24.6 | 24.5 | 22.1 | 24.9 | 25.9 | 25.4 |
| 1990 | 24.9 | 24.2 | 24.6 | 24.5 | 22.0 | 25.0 | 26.0 | 25.4 |
| 1991 | 25.0 | 24.2 | 24.7 | 24.5 | 22.0 | 25.1 | 26.2 | 25.5 |
| 1992 | 25.1 | 24.2 | 24.7 | 24.5 | 22.0 | 25.2 | 26.3 | 25.6 |
| 1993 | 25.2 | 24.2 | 24.8 | 24.6 | 22.0 | 25.3 | 26.4 | 25.7 |
| 1994 | 25.3 | 24.2 | 24.8 | 24.6 | 22.0 | 25.4 | 26.6 | 25.7 |
| 1995 | 25.4 | 24.3 | 24.9 | 24.6 | 22.0 | 25.6 | 26.7 | 25.8 |
| 1996 | 25.5 | 24.3 | 24.9 | 24.7 | 22.0 | 25.7 | 26.9 | 25.9 |
| 1997 | 25.6 | 24.3 | 25.0 | 24.7 | 22.0 | 25.8 | 27.0 | 26.0 |
| 1998 | 25.7 | 24.3 | 25.1 | 24.7 | 22.0 | 25.9 | 27.2 | 26.0 |
| 1999 | 25.8 | 24.3 | 25.1 | 24.7 | 22.0 | 26.0 | 27.3 | 26.1 |
| 2000 | 25.9 | 24.4 | 25.2 | 24.8 | 22.0 | 26.1 | 27.4 | 26.2 |
| 2001 | 25.9 | 24.4 | 25.2 | 24.8 | 21.9 | 26.2 | 27.6 | 26.3 |
| 2002 | 26.0 | 24.4 | 25.3 | 24.8 | 21.9 | 26.3 | 27.7 | 26.3 |
| 2003 | 26.1 | 24.4 | 25.3 | 24.8 | 21.9 | 26.4 | 27.8 | 26.4 |
| 2004 | 26.1 | 24.4 | 25.4 | 24.8 | 21.9 | 26.5 | 27.9 | 26.4 |
| 2005 | 26.2 | 24.4 | 25.4 | 24.9 | 21.9 | 26.5 | 28.0 | 26.5 |
| 2006 | 26.2 | 24.4 | 25.5 | 24.9 | 21.9 | 26.6 | 28.1 | 26.6 |
| 2007 | 26.3 | 24.4 | 25.5 | 24.9 | 21.9 | 26.7 | 28.2 | 26.6 |
| 2008 | 26.3 | 24.4 | 25.5 | 24.9 | 21.9 | 26.7 | 28.3 | 26.6 |
| 2009 | 26.4 | 24.4 | 25.6 | 24.9 | 21.9 | 26.8 | 28.4 | 26.7 |
| 2010 | 26.4 | 24.3 | 25.6 | 24.8 | 21.9 | 26.8 | 28.4 | 26.7 |
| 2011 | 26.4 | 24.3 | 25.7 | 24.8 | 21.9 | 26.8 | 28.5 | 26.8 |
| 2012 | 26.5 | 24.3 | 25.7 | 24.8 | 21.9 | 26.9 | 28.6 | 26.8 |
| 2013 | 26.5 | 24.3 | 25.7 | 24.8 | 21.8 | 26.9 | 28.7 | 26.8 |
| 2014 | 26.5 | 24.2 | 25.8 | 24.7 | 21.8 | 26.9 | 28.7 | 26.9 |
| 2015 | 26.6 | 24.2 | 25.8 | 24.7 | 21.8 | 27.0 | 28.8 | 26.9 |
| 2016 | 26.6 | 24.2 | 25.8 | 24.7 | 21.8 | 27.0 | 28.9 | 26.9 |USA
USA
Israel
Canada
Israel
UK
Canada
Germany
Italy
France
Germany
Italy
France
Japan
UK
Japan
Global Health Observatory data repository: Risk factors
(http://apps.who.int/gho/data/node.main.A867?lang=en)
